# The bi-directional influence of social functioning and mental health symptoms during psychological treatment: A cross-lagged analysis in young adults

**DOI:** 10.1016/j.ijchp.2025.100608

**Published:** 2025-07-05

**Authors:** Phoebe Barnett, Joshua EJ Buckman, Henry Delamain, Jae won Suh, Stephen Pilling, Rob Saunders

**Affiliations:** a CORE Data Lab, Research Department of Clinical, Educational, & Health Psychology, University College London, London, UK; b Policy Research Unit in Mental Health, Department of Psychiatry, University College London, London, UK; c iCope - Camden & Islington Psychological Therapies Services, Camden and Islington NHS Foundation Trust, London, UK; d Camden and Islington NHS Foundation Trust, London, UK; e National Collaborating Centre for Mental Health, Royal College of Psychiatrists, London, UK

**Keywords:** Young adults, Depression, Anxiety, Social functioning, Psychological therapies

## Abstract

Young adults (17–25 years old) are at greater risk of experiencing depression or anxiety, and have worse psychological therapy outcomes compared to working-age and older adults. Social functioning and related constructs are valued as outcomes of treatment, and may be particularly important to young adults, who report loneliness and a lack of social support. The relationship between social functioning and mental health during treatment in this group therefore requires further exploration. Four random intercept cross-lagged panel models were fitted to model the session-by-session change in measures of social functioning and mental health symptoms over the course of treatment among patients of NHS talking therapies for anxiety and depression services. A total of 19,600 young adults who had received at least three sessions of psychological therapy were included. There was evidence of a significant bi-directional relationship between social functioning and mental health symptoms between the third and sixth session of treatment, although associations between earlier sessions were less stable. As both mental health symptoms and social functioning were predictive of later symptom severity, further research into how support to improve social functioning could improve treatment outcomes could improve experiences of, and outcomes of treatment. Such support may also account for contextual factors relating to employment or education in this population, as some differences according to employment status emerged.

## Introduction

Young adults (aged 17–25) are at particularly high risk of experiencing mental health problems (Gustavson et al., 2018; Lu, 2019; Thapar et al., 2022). This elevated risk may stem both from transitions in life states (such as moving away from parents and other support networks) and the ongoing emotional and cognitive development occurring during this time (Arnett, 2000; Patton et al., 2016). Depression and anxiety are among the most common mental health problems experienced in this age group (Castelpietra et al., 2022). Not intervening early can result in severe and enduring consequences at the personal, societal and economic level. Experiencing a first onset of depression or anxiety during early adulthood is associated with greater odds of experiencing additional episodes or extended illness duration, as well as poor educational, marital and employment outcomes (Gustavson et al., 2018; Mojtabai et al., 2015; Ssegonja et al., 2019).

Psychological treatments are a recommended first-line intervention for depression and anxiety (NICE, 2011), however young adults present to services with higher baseline severity and symptoms improve less quickly in this group (Stochl et al., 2022). Furthermore, some young adult groups, such as those who are students or not in employment, education or training (NEET) are at even greater risk of less favourable outcomes of psychological treatments (Barnett et al., 2022; Buckman et al., 2023). Therefore, specific treatment targets which could have positive impacts on treatment response for young adults are needed.

One potential treatment target is social support, which has been argued to be crucial to support adjustment during young adulthood and as a potential protective factor against mental ill-health. Social support is associated with academic success and self-esteem at university (Conley et al., 2020) and reduced prevalence of depressive symptoms (Alsubaie et al., 2019; Sheldon et al., 2021). Therefore, it is a concern that young adults are among the most lonely (Lasgaard et al., 2016). The ability to participate actively in social activities (Timonen et al., 2021) and form close social relationships, which can contribute to development of social support networks (Alsubaie et al., 2019) collectively may represent ‘affective’ aspects of social functioning (Saris et al., 2017) alongside more objective measures such as social roles and participation in society (Burns & Patrick, 2007). These constructs are particularly valued as indicators of recovery by young adults experiencing mental health problems (McCauley et al., 2015; Pernice-Duca & Onaga, 2009), however the direction of causality between social functioning and mental health is disputed. Although some research seeking to establish the causal pathway has reported that indicators related to social functioning such as relationship conflict or loneliness are predictive of mental health symptoms (Cacioppo et al., 2010; Hawkley & Cacioppo, 2010; Özdemir & Sağkal, 2021), other work has reported that earlier depression in adolescence is associated with poor social functioning later in adulthood (Copeland et al., 2021). It is likely that the relationship between mental wellbeing and social functioning is bi-directional, meaning that each has some influence over the other (Osborn et al., 2021; Saris et al., 2017).

Understanding the bi-directional relationship between social functioning and mental health has potential to inform treatment development. For example, if social functioning is predictive of later mental health symptoms, it could illustrate a need to develop interventions which target this, supporting young adults to engage with peers and form and utilise social networks when needed. This would in turn add to the intervention options available to young adults and to the tailoring of treatment to better address their particular need, including in the reduction of loneliness. This has been identified as a critical need in young people (Eager et al., 2024). Research to date has demonstrated that during psychological treatment, social functioning at assessment is associated with treatment outcome (Buckman et al., 2021), and trajectories of improvement in social functioning are predictive of recovery in groups of young people (Barnett et al., 2023), however clarity regarding the causal nature of such associations during treatment is lacking. Cross-lagged panel models could help to delineate temporal sequences of changes in symptoms and social function over the course of psychological treatment (Falkenström et al., 2020). Identifying such temporal processes will help further our understanding of whether social support could act as a mechanism of improvement in mental health when targeted during psychological therapy, and hence if doing so could improve outcomes.

### Aims

The aim of this study was to explore the bi-directional relationship between change in social functioning and symptoms of anxiety and depression in young people receiving psychological treatment. We investigated whether fluctuations in earlier social functioning (impairment in participating in social leisure activities or developing close relationships) are associated with later depression or anxiety symptoms, or whether fluctuations in earlier depression or anxiety symptoms are associated with later social impairment.

## Method

### Participants and services

Participants were patients who attended one of eight NHS Talking Therapies services for anxiety and depression (NHS TTad, formerly known as Improving Access to Psychological Therapies (IAPT)) which are part of the North Central and East London TTad Service Improvement and Research Network (NCEL TTad SIRN) in the UK (Saunders et al., 2020). The NHS TTad services offer evidence-based psychological therapies for adults with common mental health problems within a stepped care model (Clark, 2018). Specific treatment protocols can vary across individuals, although all are recommended by the UK-based National Institute for Health and Care Excellence. These include low intensity interventions such as guided self-help and group cognitive behavioural therapy (CBT) interventions as well as high intensity interventions such as interpersonal psychotherapy or individual CBT, depending on need and preference (Clark, 2018).

The dataset consisted of participants who were referred to the services between August 2008 and August 2020. We included participants in our analysis if they:

• Were aged 17–25. There is no agreed consensus on the definition of young adulthood, however, in line with the target recipients of TTad services (adults) and World Health Organization’s definition of “young people” which gives an upper limit of this group as 24 (World Health Organisation, 2014), we chose to include participants aged between 17–25 at the point of referral to the services.
• Had individual scores for the relevant subscale items on the Work and Social Adjustment Scale (WSAS; (Mundt et al., 2002))
• Entered treatment and had data recorded for at least three treatment sessions (to sufficiently model changes during treatment)
• Scored above the cut-off for ‘caseness’ for depression or an anxiety disorder (see description in Measures)

### Measures

Two social functioning variables and two symptom severity variables were considered in longitudinal modelling:

#### Social functioning

We included two items from the Work and Social Adjustment Scale (WSAS) (Mundt et al., 2002). This measure has good psychometric properties, including high internal reliability and sensitivity to change when used in NHS TTad services (Zahra et al., 2014). Item 3 is a self-rating of the extent that a person’s mental health problem has impaired their participation in social leisure activities, such as attending parties or outings. Item 5 is a self-rating of the extent that a person’s mental health problem has impaired their ability to form and maintain close relationships. These two items were chosen and used as separate indicators of social functioning as they measure impairment in areas which may be particularly important to young people regarding forming and maintaining social support. Furthermore, as other subscales such as home management and ability to work may not be relevant to this age group, who may be living across university and home, not working, or both, use of these scales were deemed more informative than the overall WSAS score. Both of the chosen items are self-rated on a scale of 0–8, with 8 representing severe impairment and 0 representing no impairment. These items are referred to as ‘social leisure activities’ and ‘close social relationships’ throughout the rest of this paper.

#### Depressive symptoms

The Patient Health Questionnaire (PHQ-9; (Kroenke et al., 2001)) was used to measure symptoms of depression. Scores of at least 10 represent “caseness” for depression, that is, the threshold at which it is probable the respondent would meet the diagnostic criteria for a major depressive episode. The total score from the 9-item scale was used.

#### Anxiety symptoms

The Generalized Anxiety Disorder questionnaire (GAD-7; (Spitzer et al., 2006)) was used to measure symptoms of anxiety disorders. Scores of at least 8 represent “caseness” for generalized anxiety. The total score from the 7-item scale was used.

#### Covariates

As part of sensitivity analyses, the following variables were included as covariates:

1) Demographic characteristics: gender (Male, Female), age (in years), use of and prescription of medication (prescribed and taking, prescribed and not taking, not prescribed), ethnicity based on codes from UK Census (Asian, Black, Chinese, White, Mixed, Other), Indices of Multiple Deprivation (as deciles, 1–10, with 1 indicating the respondent lives in an area within the 10 % most deprived areas of residence across the country), sexual orientation (Heterosexual, Gay/Lesbian, Bisexual), employment status (employed, student, NEET, other), Long Term Conditions (LTCs; yes/no)
2) Illness severity characteristics: baseline depression (PHQ-9 score during session 1) and anxiety (GAD-7 score during session 1) symptom severity, baseline social functioning scores (WSAS scale scores during session 1), phobias recorded at baseline (yes/no), problem descriptor (diagnoses coded using the International Classification of Diseases, 10th Revisions (ICD-10), recorded during NHS TTad, which is the agreed focus of treatment between the clinician and patient and used to match patients to appropriate treatment protocols: depression, anxiety or mixed anxiety and depressive disorder diagnoses)
3) Treatment characteristics: the number of low intensity and high intensity sessions provided, time (in weeks) between referral and assessment, time (in weeks) between assessment and treatment

Where information for covariates was missing, these were imputed using Bayesian estimation in MPlus, and models estimated using 50 imputed datasets (Asparouhov & Muthén, 2010). Missing anxiety, depression or social functioning scores (from session 2 onwards) were handled using Full Information Maximum-Likelihood in Mplus.

### Statistical analysis

Random-intercept cross-lagged panel models (RI-CLPM) were estimated to explore the bi-directional relationship between fluctuations in social functioning and symptoms of anxiety or depression (Hamaker, 2018; Hamaker et al., 2015). RI-CLPMs account for “trait like” stability over time within individuals and are therefore considered a better model than traditional cross-lagged panel models (Mulder & Hamaker, 2021).

We built four models, two which explored the association between ratings of impairment in social leisure activities and depression or anxiety symptom total scores, and two which explored the association between ratings of impairment in forming close social relationships and depression or anxiety symptom total scores. In line with recent work using this data (O'Driscoll et al., 2023; Saunders et al., 2019), which indicated that the second session is usually the point that formal treatment begins (with the first session consisting primarily of assessment), we included data starting from session two and ending at session six. Session six was chosen as the endpoint because most change occurs within the first six sessions (Saunders et al., 2019).

Observed social functioning and symptom scores at each session (2–6) were used to create between-person and within-person variables. Random intercepts for each variable (one symptom measure and one social functioning measure per model) were created to represent over-arching trait-like differences in variation over time between participants using observed variables with loadings constrained to 1 (Masselink et al., 2018; Mulder & Hamaker, 2021). For variations within each person, observed social functioning and symptom scores were regressed onto their own latent factor, with resulting latent factors for each session then used to estimate autoregressive and cross-lagged paths. Residual variances of observed variables were constrained to zero to allow all variation to be captured by within- and between- person latent factors (Mulder & Hamaker, 2021).

Autoregressive paths across sessions for the same variable represented prediction of scores by scores on the same measure at the previous session, and cross-lagged paths (scores on each measure at one session and scores on the other measure at the next session) represented relationships between social functioning and symptom measures across sessions. We also accounted for correlations between the latent factor residuals at each session. This was to represent links between fluctuations in social functioning or symptoms within each person.

For the main analyses, we built four models:

1. The bi-directional relationship between impairment in social leisure activities (WSAS item 3) and depression symptoms (PHQ-9)
2. The bi-directional relationship between impairment social leisure activities (WSAS item 3) and anxiety symptoms (GAD-7)
3. The bi-directional relationship between impairment in forming close social relationships (WSAS item 5) and depression symptoms (PHQ-9)
4. The bi-directional relationship between impairment in forming close social relationships (WSAS item 5) and anxiety symptoms (GAD-7)

**Fig. 1:**
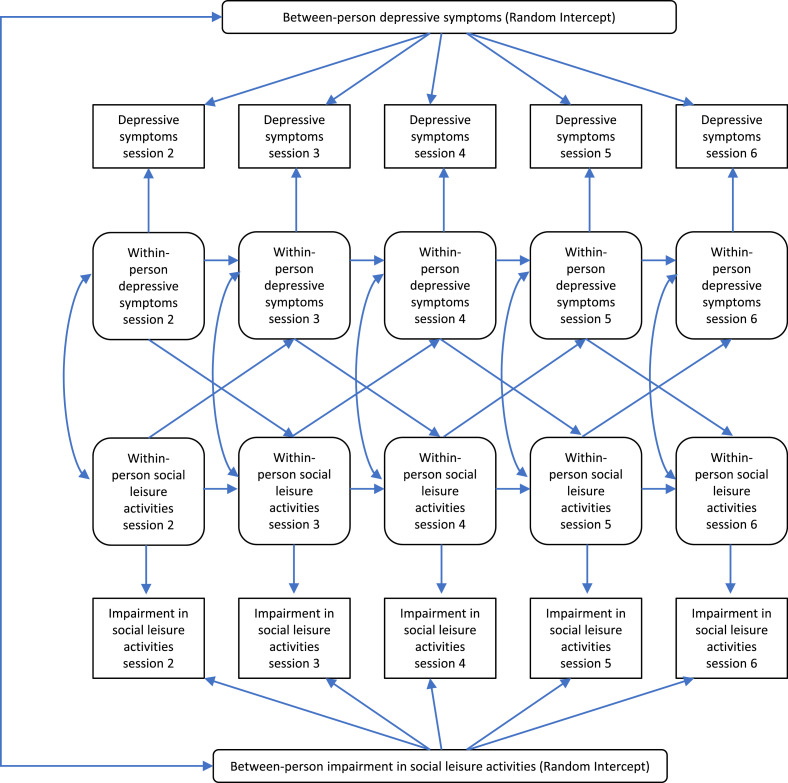
Example RI-CLPM.

The proposed model structure, using Model 1 as an example is presented in Fig. 1.

#### Sensitivity analyses

We conducted three sensitivity analyses to test the robustness of our results

1. We repeated analyses, including potential confounders as covariates in the model (see Measures).
2. We repeated analyses, including only those participants whose “problem descriptor” (see 2.2.4) matched the symptom measure being used in the model (In one model, only people treated for “depression” or “mixed anxiety and depressive disorder” were included in models using depressive symptoms as a variable (Models 1 & 3) and in another, only people with a “generalized anxiety disorder”, “OCD”, “PTSD” “Phobic anxiety or panic” or “mixed anxiety and depressive disorder” descriptor were included in models using anxiety symptoms as a variable (Models 2 & 4).
3. In line with recent research suggesting that students and people who are NEET may have worse outcomes than employed young adults (Barnett et al., 2023; Buckman et al., 2023) we repeated analyses in subgroups according to whether participants reported being i) employed, ii) in full-time education or iii) NEET (“unemployed and seeking work”, “long term sick or disabled”, “homemaker looking after the family or home who are not working”, “not receiving benefits and not working or actively seeking work” not “retired”, and not voluntary work) to explore whether findings were reflective of the young adult population as a whole.

#### Model fit

To assess model fit, we used the root mean square error of approximation (RMSEA; (Browne & Cudeck, 1992; Steiger & Lind, 1980)), the standardized root mean squared residual (SRMR; (Hu & Bentler, 1999)), the comparative fit index (CFI; (Bentler, 1990)) and the Tucker-Lewis Index (TLI; (Bentler & Bonett, 1980; Tucker & Lewis, 1973)). For the RMSEA and SRMR, values below 0.05 are indicative of excellent model fit while for the CFI and TLI, values above 0.95 are indicative of excellent fit (Byrne, 2013; Hu & Bentler, 1999).

#### Software

Stata (StataCorp, 2019) was used to clean data and compute descriptive statistics, while RI-CLPMs were constructed in Mplus Version 8 (Muthén & Muthén, 1998–2017).

## Results

From 99,621 participants aged between 17–25, we included a total of 19,600 in the final analytic sample. The participant flow diagram, including reasons for excluding participants from the sample is shown in Appendix 1.

### Descriptive statistics

Table 1 shows the descriptive statistics of the sample. The majority of participants reported they were female (72 %) and of White ethnicity (59 %). Most had “depression” as their problem descriptor (40 %), followed by GAD (16 %). On average, participants reported being “definitely” impaired in participating in social leisure activities and forming close social relationships at baseline (mean ratings of 4.44 and 4.21 out of 8 on each item, respectively). Mean scores on social functioning and symptom severity measures at each subsequent session (also see Table 1) also suggested that on average, impairment in social functioning and symptoms decreased over time. Appendix 2 provides information for each subgroup investigated in the sensitivity analyses. Average scores were similar across employed, student and NEET young adults, and across those with depression or anxiety noted as their problem descriptor.

**Table 1:** Sample baseline characteristics and treatment outcomes.

Modelled variables			
	**M**	**SD**	**n**
Baseline PHQ9^a^	15.05	5.34	19,598
Session 2 PHQ9	13.79	5.69	19,036
Session 3 PHQ9	12.67	5.86	18,956
Session 4 PHQ9	11.79	6.01	17,175
Session 5 PHQ9	11.13	6.05	15,283
Session 6 PHQ9	10.67	6.04	13,368
Baseline GAD7^a^	13.93	4.3	19,592
Session 2 GAD7	12.89	4.85	19,028
Session 3 GAD7	11.83	5.11	18,950
Session 4 GAD7	11.01	5.24	17,173
Session 5 GAD7	10.37	5.33	15,279
Session 6 GAD7	9.88	5.35	13,365
Baseline WSAS-3 (social leisure activities) a	4.44	2.31	18,105
Session 2 WSAS-3	4.15	2.29	18,011
Session 3 WSAS-3	3.87	2.28	17,905
Session 4 WSAS-3	3.65	2.28	16,288
Session 5 WSAS-3	3.46	2.27	14,515
Session 6 WSAS-3	3.35	2.25	12,729
Baseline WSAS-5 (close relationships) a	4.21	2.4	18,098
Session 2 WSAS-5	3.94	2.33	18,006
Session 3 WSAS-5	3.68	2.3	17,908
Session 4 WSAS-5	3.48	2.28	16,286
Session 5 WSAS-5	3.3	2.27	14,514
Session 6 WSAS-5	3.2	2.27	12,728
Other baseline variables			
Continuous variables	**M**	**SD**	**n**
Baseline WSAS item 1 (employment)	5.16	2.79	18,078
Baseline WSAS-2 (home management)	3.43	2.38	18,106
Baseline WSAS-4 (private leisure)	3.57	2.5	18,097
Agoraphobia Item	2.92	2.66	19,407
Social Phobia Item	3.45	2.48	19,410
Specific Phobia Item	2.3	2.63	19,408
Number of low intensity sessions	2.93	2.74	19,600
Number high intensity sessions	5.33	5.47	19,600
Number total sessions	8.31	4.7	19,600
Weeks between referral to assessment^b^	3.29	3.44	19,593
Weeks between assessment to treatment^b^	8.84	8.33	18,953
Age	22.17	2.28	19,600
Categorical variables		**N**	**%**
Gender	Male	5496	28.04
	Female	14,027	71.57
	Missing	77	0.39
Ethnicity	White	11,606	59.21
	Mixed	1647	8.4
	Asian	2371	12.1
	Black	2346	11.97
	Chinese	211	1.08
	Other	614	3.13
	Missing	805	4.11
Employment status	Employed	10,001	51.03
	Student	5215	26.61
	NEET	6061	20.72
	Other (voluntary work or missing)	323	1.64
IMD Decile	1	1877	9.58
	2	5421	27.66
	3	4082	20.83
	4	2372	12.1
	5	1795	9.16
	6	1459	7.44
	7	930	4.74
	8	840	4.29
	9	374	1.91
	10	151	0.77
	Missing	299	1.53
Sexual orientation	Heterosexual	13,265	67.68
	Gay/Lesbian	592	3.02
	Bi-sexual	903	4.61
	Missing	4840	24.69
Medication	Prescribed not taking	881	4.49
	Prescribed and taking	5079	25.91
	Not prescribed	12,347	62.99
	Missing	1293	6.6
Long term condition	No	12,949	66.07
	Yes	2910	14.85
	Missing	3741	19.09
Problem descriptor	Depression	7849	40.05
	Mixed anxiety and depressive disorder	1159	5.91
	GAD	3104	15.84
	OCD	617	3.15
	PTSD	679	3.46
	Other Phobia & Panic	1327	6.77
	Social Phobia	1201	6.13
	Unspecified anxiety	809	4.13
	Missing	2855	14.57
Clinical outcomes^c^	Reliable recovery	9244	47.16
	Reliable improvement	14,143	72.16
	Deterioration	1294	6.6
	Attrition	5792	31.9

a Baseline variables were only used in sensitivity analyses to compute RI-CLPMs.

b Winsorized at the top 99 % to remove extreme variables.

c Reliable recovery: moving from “caseness” to “non-caseness” and reporting reliable improvement. Reliable improvement/deterioration: reporting a reduction/increase in symptom scores larger than the reliable change threshold. Attrition: dropping out of the episode of care before completing planned treatment sessions. For more information see National Collaborating Centre for Mental Health (2024).

### Model fit

For each of the Models 1–4, we observed excellent measures of model fit. These are shown in Table 2.

**Table 2:** Model fit statistics for main models.

Model	RMSEA	CFI	TLI	SRMR
1 (Social leisure activities, depressive symptoms)	0.036	0.993	0.986	0.021
2 (Social leisure activities, anxiety symptoms)	0.04	0.992	0.982	0.023
3 (Close social relationships, depressive symptoms)	0.037	0.993	0.985	0.023
4 (Close social relationships, depressive symptoms)	0.039	0.992	0.982	0.024

RMSEA: Root Mean Square Error of Approximation, CFI: Comparative Fit Index, TLI: Tucker-Lewis Index, SRMR: Standardized Root Mean squared Residual.

### Correlations

Correlations between symptom and functioning measures at each timepoint increased over time. For example, in Model 1, the correlation between impairment in social leisure activities and depressive symptoms increased from 0.417 during the second session to 0.578 during the sixth, with other models demonstrating similar increases. This suggested that within each individual, improvement in one facet was associated with improvement in the other.

### Autoregressive and cross-lagged paths

In all four primary models, all autoregressive paths were significant, suggesting that scores on an individual measure during one session were predictive of scores on that measure during the next session.

In addition, in all models, there were significant cross-lagged paths in both directions from session 3 onwards indicating a bi-directional relationship between mental health symptoms and social functioning. This suggested that between session 3 and 6, an individual’s self-rated impairment in participation in social leisure activities and forming close social relations was predictive of the severity of their depression and anxiety symptoms in future treatment sessions. Similarly, symptom severity was predictive of future levels of impairment in social functioning.

Before the third session, there was a negative coefficient between measures of social functioning at session 2 and depression or anxiety symptoms at session 3. Although depressive symptoms at session 2 were associated with social functioning at session 3 (*p* < 0.001), anxiety symptoms at session 2 were not significantly associated with either measure of social functioning at session 3 (*p* = 0.313 and *p* = 0.357, respectively for social leisure activities (Model 2) and close social relationships (Model 4). Table 3 provides all results of the main analyses. See Appendix 3 for visual diagrams of associations.

**Table 3:** Autoregressive and cross-lagged paths for the main analysis (*n* = 19,600).

			1 (PHQ9, WSAS 3)		2 (GAD7, WSAS3)		3 (PHQ9, WSAS5)		4 (GAD7, WSAS5)	
Path	**Predictor**	**Outcome**	**Standardized coefficient**	**p-value**	**Standardized coefficient**	**p-value**	**Standardized coefficient**	**p-value**	**Standardized coefficient**	**p-value**
Autoregressive	WSAS measure S2	WSAS measure S3	0.129	<0.001	0.133	<0.001	0.126	<0.001	0.136	<0.001
	WSAS measure S3	WSAS measure S4	0.154	<0.001	0.172	<0.001	0.171	<0.001	0.183	<0.001
	WSAS measure S4	WSAS measure S5	0.206	<0.001	0.217	<0.001	0.207	<0.001	0.208	<0.001
	WSAS measure S5	WSAS measure S6	0.265	<0.001	0.272	<0.001	0.273	<0.001	0.271	<0.001
	Symptom measure S2	Symptom measure S3	0.294	<0.001	0.273	<0.001	0.290	<0.001	0.263	<0.001
	Symptom measure S3	Symptom measure S4	0.342	<0.001	0.374	<0.001	0.343	<0.001	0.348	<0.001
	Symptom measure S4	Symptom measure S5	0.385	<0.001	0.435	<0.001	0.397	<0.001	0.413	<0.001
	Symptom measure S5	Symptom measure S6	0.429	<0.001	0.460	<0.001	0.447	<0.001	0.453	<0.001
Cross-lagged	WSAS measure S2	PHQ9 S3	−0.037	0.003	−0.064	0.015	−0.031	0.008	−0.037	0.002
	WSAS measure S3	PHQ9 S4	0.342	<0.001	0.170	<0.001	0.033	0.009	0.051	<0.001
	WSAS measure S4	PHQ9 S5	0.123	<0.001	0.308	<0.001	0.085	<0.001	0.107	<0.001
	WSAS measure S5	PHQ9 S6	0.128	<0.001	0.358	<0.001	0.097	<0.001	0.120	<0.001
	Symptom measure S2	WSAS measure S3	0.053	<0.001	0.007	0.313	0.051	<0.001	0.013	0.357
	Symptom measure S3	WSAS measure S4	0.127	<0.001	0.061	<0.001	0.102	<0.001	0.127	<0.001
	Symptom measure S4	WSAS measure S5	0.221	<0.001	0.105	<0.001	0.166	<0.001	0.209	<0.001
	Symptom measure S5	WSAS measure S6	0.242	<0.001	0.106	<0.001	0.219	<0.001	0.235	<0.001

WSAS: Work and Social Adjustment Scale. Symptom measures: PHQ9: Patient Health Questionnaire, GAD7: General Anxiety Disorder Questionnaire. S: Session.

### Sensitivity analyses

Three sensitivity analyses were conducted to explore robustness of results. We also found excellent model fit for these models with broadly similar results across the RMSEA, CFI, TFI and SRMR. Full details of model fit for all models are available in Appendix 4.

#### Covariates

Covariates (including demographic, baseline severity and treatment-related factors) were added to all four models (see Appendix 5). Models were similar to primary analyses between sessions 3–6 with all associations continuing to be significant. Between session 2 and 3 the addition of covariates altered some associations between variables: Only impairment in close social relationships continued to predict reduced anxiety symptoms at session 3. Anxiety at session 2 significantly predicted increased impairment in social leisure activities at session 3 after adjusting for covariates. Finally, depressive symptoms at session 2 no longer significantly predicted increased impairment in close social relationships at session 3. Overall, the results suggested that associations between sessions 3–6 were robust.

#### Problem descriptor

In the second sensitivity analysis, only those provided with a problem descriptor matching the symptom measure being modelled were included in the analysis. Differences were once again only seen in paths between measures at session 2 and measures at session 3, such that in those with either depression or mixed anxiety and depressive disorder, higher impairment in social leisure activities at session 2 did not predict higher depression scores at session 3, and in those with either anxiety or mixed anxiety and depressive disorder, neither impairment in social leisure activities nor impairment in close social relationships at session 2 predicted anxiety scores at session 3. Also, in those with depression or mixed anxiety and depressive disorder, depression scores at session 2 no longer predicted impairment in close social relationships at session 3. Full details of results can be found in Appendix 6.

#### Employment status

In the third sensitivity analysis, we explored differences in relationships between variables stratified by employment status (employed, student, NEET). We found that although few differences in relationships between variables existed between young adults in employment compared to education, relationships between session 2 and session 3 variables were weaker in young people who were NEET, such that autoregressive associations between social functioning measures at session 2 and 3 were not significant in those who were NEET, and cross-lagged associations between depression symptoms at session 2 did not predict either social functioning measure at session 3 in those who were NEET. However, it was only in NEETs, and not students or those who were employed, where higher social functioning impairment measures at session 2 predicted lower anxiety or depression symptoms at session 3. We also found that associations between session 3 measures of impairment in close social relationships and session 4 measures of anxiety or depression were primarily driven by students. Full details of results can be found in Appendix 6.

## Discussion

This study explored associations between measures of social functioning and symptom severity over the course of psychological treatment. Findings suggest that there is a bidirectional relationship between social functioning and mental health symptoms between the third and sixth session of psychological treatment in NHS TTad services. Sensitivity analyses further supported findings, although they also highlighted instability in cross-lagged associations between the second and third session. This may illustrate that the third session onwards is the point that the main treatment protocol is taking effect, and potentially the point that social functioning is likely to be used as a therapeutic tool, for example planning activities during behavioural activation. Previous research (Saunders et al., 2023) exploring cross-lagged associations between depressive symptoms and sleep has also noted that these tend to emerge from session 3, supporting the current findings and indicating that measures taken earlier in the treatment process may be less indicative of transdiagnostic improvement across measurements in later sessions, although they may still predict improvement within domains.

We found that generally, coefficients for the relationship between earlier anxiety or depression and later social functioning were larger than vice versa. This supports evidence that symptoms are particularly predictive of later social functioning in young adults (Copeland et al., 2021) as well as older adults (McHugh Power et al., 2020). However, as this analysis followed associations over the course of treatment, which, in routine psychological services tends to target the experienced symptoms over goals relating to wider social determinants and community activity (Porter et al., 2024), it is possible that changes over time in symptoms were less consistent than vice versa, and therefore less easily predicted by social functioning measures. However, sensitivity analyses including only those whose problem descriptor matched the measured symptoms, which would have exacerbated this effect, did not support this hypothesis.

The presence of a bi-directional association between sessions 3–6 does however imply that provision of additional support with social functioning alongside psychological therapy could lead to future improvements in both social functioning and symptoms. Provision of such support could follow previous examples targeting other aspects of social functioning, which have shown positive effects on outcomes. For example, support with employment through embedding employment advisors in psychological treatment programmes, which improved employment outcomes and also some mental health outcomes above standard care (Hogarth et al., 2013), and enhancing services with a health and wellbeing pathway to combat wider determinants of mental health, which led to small improvements in mental health alongside positive qualitative feedback from patients (Porter et al., 2024). As social outcomes are particularly favoured by young adults (McCauley et al., 2015; Pernice-Duca & Onaga, 2009), further randomized controlled trials exploring how embedded support with participation in social activities or forming/maintaining close social relationships could impact qualitative and quantitative indicators of intervention effectiveness.

Sensitivity analyses demonstrated that young adults who are NEET are particularly different from students or employed adults in the extent that session 2 measures can predict session 3 measures. Young people who are NEET represent a particularly vulnerable group who tend to have less positive outcomes in NHS TTad services (Buckman et al., 2023), and the lack of association between earlier symptoms or social functioning and later social functioning may highlight that there is less transdiagnostic transfer of symptoms or social functioning across domains in this group, necessitating more holistic support early in the treatment process to support their recovery.

### Limitations

While this analysis contributes to the available evidence regarding links between social functioning and mental health in young adults, some important limitations should be acknowledged. Although in the TTad dataset, the WSAS is currently the best available measure of social functioning, as items ask participants to self-report the level of impairment in social aspects *as a result* of mental health symptoms, it is possible that the association is already biased to be stronger for symptoms preceding social functioning. Analyses also used individual items from the total measure, limiting available variance in this construct. It is possible that an alternative measure which does not conflate social functioning and impacts of symptoms of mental health problems would allow for further exploration of the separate impact of such social activities on mental health. This analysis used the GAD-7 and PHQ-9 to measure anxiety and depression symptoms, respectively. These measures are often used as self-reported screening tools and therefore there is a risk that their use as symptom severity measures limits the clinical application of results. However, these measures have been shown to be more sensitive to change than clinician rated scales (Kounali et al., 2016). Furthermore, we only used GAD-7 as a measure of anxiety symptoms, although this may not be sensitive to all anxiety disorders and their symptoms (Beard & Björgvinsson, 2014).

In addition, although sensitivity analyses controlled for the number of low and high intensity treatment sessions received (with some observed differences), it is important to note that analyses did not differentiate different forms of treatment provided nor were there measures of fidelity to treatment protocols. This means that some participants may have received more support with their difficulties participating in social activities than others as part of treatment, for example behavioural activation techniques may be particularly helpful in encouraging participation if they were so focused. Some participants may also have received sessions more or less frequently than standard NICE-recommended guidance suggesting that sessions be provided weekly (National Collaborating Centre for Mental Health, 2024) Similarly, we were also unable to consider other forms of support or intervention that participants may have utilized outside of TTad services, or the availability of social support for each patient (for example, whether young people are living at home or have moved away, or have a long-term partner), further adding to difficulties reflecting the variety of interventions that may have been received by participants.

Finally, although real-world evidence provides a number of strengths to support treatment development (Liu & Panagiotakos, 2022), its use in this study also limits its ability to further explore relationships between confounding variables and outcomes. Use of routinely collected data also can be subject to selection bias, for example disparities between ethnic groups in terms of access and effectiveness have been reported (Arundell et al., 2024).

### Implications for research and practice

The bi-directional relationship between measures of social functioning and symptom severity illustrated in this study suggests that alongside symptom-specific treatment targets, there may be an additional benefit of supporting young people with their social relationships and participation in social activities to improve both symptom and social functioning outcomes. However, the limitations of this study also necessitate further work to build on the findings outlined here. It will be important to explore whether the associations reported here remain the same if an alternative measure of social support is used. Using another measure would help to understand whether current associations are a bi-product of the WSAS items targeting symptom-induced impairment in social functioning. This is particularly important as negative self-evaluation has been reported to be associated with depressive symptoms in adolescents (Hards et al., 2020). More objective measures of social support and functioning may therefore be required.

Furthermore, although in this study we did not have information regarding specific treatment interventions used in session or fidelity to treatment, there is scope to gain a more robust understanding of whether the relationships between social functioning and mental health are stable or dynamic according to interventions used through a clinical trial. For example, differences in associations may arise when interventions which have a more specific focus on the symptoms of depression or anxiety (for example, rumination) are utilised compared to more transdiagnostic support such as behavioural activation. There is at present also a lack of research which focuses specifically on effectiveness of integrating additional targeted support with social relationships for young adults using mental health services compared to standard care, and therefore further work is required to understand whether there are additive benefits of this for recovery. However, previous research has suggested that interventions most successful in treating social isolation and loneliness are targeted at the specific needs of the population in specific contexts (Osborn et al., 2021), and therefore future research could also explore how local community services can facilitate social connection, as is currently being explored for people with treatment-resistant anxiety and depression (Stefanidou et al., 2023). Furthermore, It is important to ensure that young people are provided with support which is appropriate for their needs and which they consider acceptable (Eager et al., 2024), which may require further adaptation according to employment status and living situations.

### Conclusion

Overall, a bidirectional relationship exists between social functioning and mental health over the course of psychological therapy in young adults, although this relationship is less clear in the early stages of treatment. The presence of an association between social functioning and later mental health, supports an argument for further consideration into whether embedding support with social relationships and activity for young adults could improve outcomes of psychological therapy for this group. Further research into how such support could adequately account for contextual factors and preferred outcomes of young adults is also required.

## Declaration of competing interest

The authors declare that they have no known competing financial interests or personal relationships that could have appeared to influence the work reported in this paper.

## Appendix Supplementary materials

Image, application 1

Image, application 2

Image, application 3

Image, application 4

Image, application 5

Image, application 6

Image, application 7
