# Supplementary material for: The bi-directional influence of social functioning and mental health symptoms during psychological treatment: A cross-lagged analysis in young adults
Source: Int J Clin Health Psychol. 2025 Jul 5;25(3):100608. doi: 10.1016/j.ijchp.2025.100608 (PMC12272429; doi:10.1016/j.ijchp.2025.100608)
Supplement: Supplementary file 1 [file mmc1.docx]

# Appendix 1: Participant flow diagram

99,621 referrals of participants aged between 17-25

Did not enter treatment and have at least 3 sessions

Did not enter treatment: 71437 participants excluded

Had less than 3 sessions: 3695 participants excluded

24,489 participants remaining

Scoring below “caseness” for anxiety and depression: 1,987 participants excluded

22,502 participants remaining

No individual scores available on Work and Social Adjustment Scale items: 2898 participants excluded

No scores available for sessions 2-6: 4 participants excluded

19,600 participants remaining
