# Supplementary material for: The bi-directional influence of social functioning and mental health symptoms during psychological treatment: A cross-lagged analysis in young adults
Source: Int J Clin Health Psychol. 2025 Jul 5;25(3):100608. doi: 10.1016/j.ijchp.2025.100608 (PMC12272429; doi:10.1016/j.ijchp.2025.100608)
Supplement: Supplementary file 2 [file mmc2.docx]

# Appendix 2: Baseline characteristics by sub-group

## 2.1: Problem descriptor

| Modelled variables | | | | | | | | | |
| --- | --- | --- | --- | --- | --- | --- | --- | --- | --- |
|  | | **Anxiety or mixed anxiety and depression^b^** | | | **Depression or mixed anxiety and depression^b^** | | | | |
|  | | **M** | **SD** | **n** | **M** | | **SD** | | **n** |
| Baseline PHQ9^a^ | | 13.84 | 5.55 | 8,408 | 16.48 | | 4.77 | | 9,008 |
| Session 2 PHQ9 | | 12.79 | 5.76 | 8,198 | 15.14 | | 5.35 | | 8,780 |
| Session 3 PHQ9 | | 11.88 | 5.92 | 8,161 | 13.82 | | 5.68 | | 8,760 |
| Session 4 PHQ9 | | 11.05 | 6.01 | 7,508 | 12.88 | | 5.92 | | 7,835 |
| Session 5 PHQ9 | | 10.45 | 5.99 | 6,799 | 12.23 | | 6.05 | | 6,928 |
| Session 6 PHQ9 | | 10.06 | 6 | 5,965 | 11.7 | | 6.08 | | 6,072 |
| Baseline GAD7^a^ | | 14.5 | 4.08 | 8,406 | 13.63 | | 4.42 | | 9,004 |
| Session 2 GAD7 | | 13.34 | 4.72 | 8,198 | 12.81 | | 4.89 | | 8,771 |
| Session 3 GAD7 | | 12.25 | 5.05 | 8,160 | 11.82 | | 5.13 | | 8,758 |
| Session 4 GAD7 | | 11.34 | 5.19 | 7,511 | 11.06 | | 5.29 | | 7,831 |
| Session 5 GAD7 | | 10.58 | 5.3 | 6,797 | 10.53 | | 5.34 | | 6,927 |
| Session 6 GAD7 | | 10.13 | 5.36 | 5,967 | 10.04 | | 5.33 | | 6,068 |
| Baseline WSAS-3^a^ | | 4.34 | 2.34 | 7,820 | 4.63 | | 2.28 | | 8,421 |
| Session 2 WSAS-3 | | 4.09 | 2.31 | 7,916 | 4.33 | | 2.29 | | 8,275 |
| Session 3 WSAS-3 | | 3.85 | 2.29 | 7,774 | 4.03 | | 2.28 | | 8,335 |
| Session 4 WSAS-3 | | 3.61 | 2.31 | 7,195 | 3.79 | | 2.27 | | 7,461 |
| Session 5 WSAS-3 | | 3.44 | 2.28 | 6,494 | 3.61 | | 2.28 | | 6,594 |
| Session 6 WSAS-3 | | 3.33 | 2.27 | 5,744 | 3.5 | | 2.26 | | 5,777 |
| Baseline WSAS-5^a^ | | 3.91 | 2.43 | 7,819 | 4.61 | | 2.33 | | 8,419 |
| Session 2 WSAS-5 | | 3.67 | 2.36 | 7,911 | 4.32 | | 2.29 | | 8,275 |
| Session 3 WSAS-5 | | 3.46 | 2.31 | 7,775 | 4.01 | | 2.28 | | 8,335 |
| Session 4 WSAS-5 | | 3.27 | 2.29 | 7,194 | 3.79 | | 2.27 | | 7,460 |
| Session 5 WSAS-5 | | 3.1 | 2.26 | 6,493 | 3.63 | | 2.27 | | 6,590 |
| Session 6 WSAS-5 | | 3 | 2.26 | 5,744 | 3.52 | | 2.28 | | 5,778 |
| Other baseline variables | | | | | | | | | |
|  | | **Anxiety or mixed anxiety and depression^b^** | | | **Depression or mixed anxiety and depression^b^** | | | | |
| Continuous variables | | **M** | **SD** | **n** | **M** | | **SD** | | **n** |
| Baseline WSAS-1 | | 5.02 | 2.81 | 7,811 | 5.43 | | 2.74 | | 8,404 |
| Baseline WSAS-2 | | 3.11 | 2.36 | 7,820 | 3.79 | | 2.37 | | 8,422 |
| Baseline WSAS-4 | | 3.32 | 2.5 | 7,818 | 3.88 | | 2.49 | | 8,419 |
| Agoraphobia Item | | 3.29 | 2.71 | 8,347 | 2.68 | | 2.59 | | 8,909 |
| Social Phobia Item | | 3.45 | 2.51 | 8,349 | 3.55 | | 2.47 | | 8,909 |
| Specific Phobia Item | | 2.55 | 2.71 | 8,349 | 2.12 | | 2.56 | | 8,909 |
| Number LI sessions | | 2.83 | 2.7 | 8,410 | 5.09 | | 5.43 | | 9,008 |
| Number HI sessions | | 5.8 | 5.62 | 8,410 | 3.1 | | 2.8 | | 9,008 |
| Number total sessions | | 8.68 | 4.79 | 8,410 | 8.24 | | 4.78 | | 9,008 |
| Weeks-referral to assessment | | 3.38 | 3.62 | 8,410 | 3.13 | | 3.31 | | 9,006 |
| Weeks- assessment to treatment | | 8.79 | 8.41 | 8,151 | 9.09 | | 8.22 | | 8,643 |
| Age | | 22.21 | 2.3 | 8410 | 22.11 | | 2.27 | | 9008 |
|  | |  | **Anxiety or mixed anxiety and depression^b^** | | | **Depression or mixed anxiety and depression^b^** | | | |
| Categorical variables | | | **N** | **%** | | **N** | | **%** | |
| Gender | Male | | 2391 | 28.43 | | 2480 | | 27.53 | |
|  | Female | | 5997 | 71.31 | | 6498 | | 72.14 | |
|  | Missing | | 22 | 0.26 | | 30 | | 0.33 | |
| Ethnicity | White | | 5429 | 64.55 | | 4865 | | 54.01 | |
|  | Mixed | | 681 | 8.1 | | 818 | | 9.08 | |
|  | Asian | | 844 | 10.04 | | 1400 | | 15.54 | |
|  | Black | | 831 | 9.88 | | 1289 | | 14.31 | |
|  | Chinese | | 82 | 0.98 | | 101 | | 1.12 | |
|  | Other | | 261 | 3.1 | | 294 | | 3.26 | |
|  | Missing | | 282 | 3.35 | | 241 | | 2.68 | |
| IMD Decile | 1 | | 828 | 9.85 | | 839 | | 9.31 | |
|  | 2 | | 2363 | 28.1 | | 2451 | | 27.21 | |
|  | 3 | | 1760 | 20.93 | | 1831 | | 20.33 | |
|  | 4 | | 985 | 11.71 | | 1137 | | 12.62 | |
|  | 5 | | 759 | 9.02 | | 841 | | 9.34 | |
|  | 6 | | 598 | 7.11 | | 712 | | 7.9 | |
|  | 7 | | 396 | 4.71 | | 420 | | 4.66 | |
|  | 8 | | 379 | 4.51 | | 383 | | 4.25 | |
|  | 9 | | 171 | 2.03 | | 174 | | 1.93 | |
|  | 10 | | 58 | 0.69 | | 72 | | 0.8 | |
|  | Missing | | 113 | 1.34 | | 148 | | 1.64 | |
| Sexual orientation | Heterosexual | | 5802 | 68.99 | | 6379 | | 70.81 | |
|  | Gay/Lesbian | | 263 | 3.13 | | 273 | | 3.03 | |
|  | Bi-sexual | | 326 | 3.88 | | 472 | | 5.24 | |
|  | Missing | | 2019 | 24.01 | | 1884 | | 20.91 | |
| Medication | Prescribed not taking | | 414 | 4.92 | | 378 | | 4.2 | |
|  | Prescribed and taking | | 2014 | 23.95 | | 2514 | | 27.91 | |
|  | Not prescribed | | 5503 | 65.43 | | 5485 | | 60.89 | |
|  | Missing | | 479 | 5.7 | | 631 | | 7 | |
| Long term condition | No | | 5599 | 66.58 | | 6019 | | 66.82 | |
|  | Yes | | 1225 | 14.57 | | 1420 | | 15.76 | |
|  | Missing | | 1586 | 18.86 | | 1569 | | 17.42 | |
| Problem descriptor | Depression | | 0 | 0 | | 7849 | | 87.13 | |
|  | Mixed A.D | | 1159 | 13.78 | | 1159 | | 12.87 | |
|  | GAD | | 3104 | 36.91 | | 0 | | 0 | |
|  | OCD | | 617 | 7.34 | | 0 | | 0 | |
|  | PTSD | | 679 | 8.07 | | 0 | | 0 | |
|  | Other Phobia & Panic | | 1327 | 15.78 | | 0 | | 0 | |
|  | Social Phobia | | 1201 | 14.28 | | 0 | | 0 | |
|  | Unspecified anxiety | | 323 | 3.84 | | 0 | | 0 | |
|  | Missing | | 0 | 0 | | 0 | | 0 | |
| Clinical outcomes | Reliable recovery | | 4073 | 48.43 | | 4054 | | 45 | |
|  | Reliable improvement | | 6237 | 74.16 | | 6328 | | 70.25 | |
|  | Deterioration | | 451 | 5.36 | | 703 | | 7.8 | |
|  | Attrition | | 2310 | 29.45 | | 2902 | | 35.09 | |
| ^a^ baseline variables were only used in sensitivity analyses to compute RI-CLPMs | | | | | | | | | |
| ^b^ Participants with mixed anxiety and depression diagnoses are represented in both groups | | | | | | | | | |
| WSAS: Work and Social Adjustment Scale. LI: Low intensity. HI: High intensity. A.D. Anxiety and Depression. GAD: Generalized Anxiety Disorder. OCD: Obsessive Compulsive Disorder. PTSD: Post-Traumatic Stress Disorder | | | | | | | | | |

## 2.2: Employment status

| Modelled variables | | | | | | | | | | | | | | |
| --- | --- | --- | --- | --- | --- | --- | --- | --- | --- | --- | --- | --- | --- | --- |
|  | | **Employed** | | | **Student** | | | | **NEET** | | | | | |
|  | | **M** | **SD** | **n** | **M** | | **SD** | | **n** | **M** | | **SD** | | **n** |
| Baseline PHQ9^a^ | | 14.47 | 5.36 | 10,001 | 15.09 | | 5.21 | | 5,214 | 16.41 | | 5.2 | | 4,061 |
| Session 2 PHQ9 | | 13.13 | 5.61 | 9,717 | 13.84 | | 5.57 | | 5,072 | 15.38 | | 5.69 | | 3,949 |
| Session 3 PHQ9 | | 11.94 | 6 | 9,675 | 12.86 | | 6 | | 5,040 | 14.25 | | 6 | | 3,933 |
| Session 4 PHQ9 | | 11.08 | 6 | 8,821 | 12.01 | | 6 | | 4,593 | 13.31 | | 6 | | 3,491 |
| Session 5 PHQ9 | | 10.43 | 6 | 7,921 | 11.36 | | 6 | | 4,128 | 12.66 | | 6 | | 2,997 |
| Session 6 PHQ9 | | 9.97 | 5.79 | 6,921 | 10.86 | | 5.98 | | 3,612 | 12.17 | | 6 | | 2,624 |
| Baseline GAD7^a^ | | 13.92 | 4.24 | 9,999 | 13.62 | | 4.27 | | 5,213 | 14.35 | | 4.44 | | 4,059 |
| Session 2 GAD7 | | 12.76 | 4.81 | 9,710 | 12.54 | | 4.77 | | 5,069 | 13.65 | | 4.94 | | 3,950 |
| Session 3 GAD7 | | 11.58 | 5.07 | 9,675 | 11.62 | | 5 | | 5,035 | 12.73 | | 5.23 | | 3,932 |
| Session 4 GAD7 | | 10.7 | 5 | 8,824 | 10.87 | | 5 | | 4,586 | 12 | | 5 | | 3,492 |
| Session 5 GAD7 | | 10 | 5 | 7,918 | 10.3 | | 5 | | 4,128 | 11.44 | | 6 | | 2,996 |
| Session 6 GAD7 | | 9.48 | 5.22 | 6,918 | 9.84 | | 5.26 | | 3,613 | 10.96 | | 6 | | 2,623 |
| Baseline WSAS-3^a^ | | 4.27 | 2.25 | 9,210 | 4.4 | | 2.25 | | 4,888 | 4.94 | | 2.47 | | 3,772 |
| Session 2 WSAS-3 | | 3.94 | 2.21 | 9,207 | 4.16 | | 2.22 | | 4,833 | 4.65 | | 2.47 | | 3,720 |
| Session 3 WSAS-3 | | 3.62 | 2 | 9,114 | 3.94 | | 2 | | 4,806 | 4.38 | | 2 | | 3,714 |
| Session 4 WSAS-3 | | 3.42 | 2 | 8,342 | 3.67 | | 2 | | 4,386 | 4.19 | | 2 | | 3,318 |
| Session 5 WSAS-3 | | 3.23 | 2 | 7,483 | 3.52 | | 2 | | 3,966 | 3.97 | | 3 | | 2,853 |
| Session 6 WSAS-3 | | 3.1 | 2.15 | 6,552 | 3.43 | | 2.21 | | 3,490 | 3.88 | | 2 | | 2,493 |
| Baseline WSAS-5^a^ | | 4.15 | 2.37 | 9,208 | 4.16 | | 2.36 | | 4,888 | 4.41 | | 2.52 | | 3,767 |
| Session 2 WSAS-5 | | 3.87 | 2.3 | 9,201 | 3.9 | | 2.3 | | 4,834 | 4.17 | | 2.44 | | 3,720 |
| Session 3 WSAS-5 | | 3.58 | 2 | 9,113 | 3.67 | | 2 | | 4,807 | 3.94 | | 2 | | 3,717 |
| Session 4 WSAS-5 | | 3.36 | 2 | 8,342 | 3.49 | | 2 | | 4,385 | 3.77 | | 2 | | 3,317 |
| Session 5 WSAS-5 | | 3.15 | 2 | 7,483 | 3.31 | | 2 | | 3,965 | 3.65 | | 2 | | 2,853 |
| Session 6 WSAS-5 | | 3.07 | 2.21 | 6,551 | 3.21 | | 2.24 | | 3,490 | 3.53 | | 2.42 | | 2,493 |
| Other baseline variables | | | | | | | | | | | | | | |
|  | | **Employed** | | | **Student** | | | | **NEET** | | | | | |
| Continuous variables | | **M** | **SD** | **n** | **M** | | **SD** | | **n** | **M** | | **SD** | | **n** |
| Baseline WSAS-1 | | 4.26 | 2.4 | 9,203 | 5.65 | | 2.81 | | 4,876 | 6.74 | | 2.75 | | 3,765 |
| Baseline WSAS-2 | | 3.34 | 2.32 | 9,212 | 3.36 | | 2.33 | | 4,888 | 3.74 | | 2.53 | | 3,771 |
| Baseline WSAS-4 | | 3.49 | 2.46 | 9,207 | 3.54 | | 2.42 | | 4,887 | 3.82 | | 2.66 | | 3,768 |
| Agoraphobia Item | | 2.7 | 2.58 | 9,906 | 2.97 | | 2.6 | | 5,184 | 3.42 | | 2.83 | | 4,019 |
| Social Phobia Item | | 3.14 | 2.39 | 9,908 | 3.57 | | 2.42 | | 5,184 | 4.04 | | 2.63 | | 4,020 |
| Specific Phobia Item | | 2.06 | 2.51 | 9,907 | 2.38 | | 2.58 | | 5,183 | 2.78 | | 2.88 | | 4,020 |
| Number LI sessions | | 3.02 | 2.75 | 10,001 | 2.96 | | 2.78 | | 5,215 | 2.66 | | 2.66 | | 4,061 |
| Number HI sessions | | 5.35 | 5.57 | 10,001 | 5.25 | | 5.41 | | 5,215 | 5.42 | | 5.31 | | 4,061 |
| Number total sessions | | 8.42 | 4.74 | 10,001 | 8.25 | | 4.59 | | 5,215 | 8.14 | | 4.77 | | 4,061 |
| Weeks-referral to assessment | | 3.23 | 3.4 | 10,000 | 3.37 | | 3.31 | | 5,211 | 3.31 | | 3.67 | | 4,061 |
| Weeks- assessment to treatment | | 9.05 | 8.41 | 9,693 | 8.43 | | 8.01 | | 5,072 | 8.92 | | 8.43 | | 3,892 |
| Age | | 22.99 | 1.9 | 10,001 | 20.64 | | 2.2 | | 5,215 | 22.1 | | 2.2 | | 4,061 |
|  |  | | **Employed** | | | **Student** | | | | | **NEET** | | | |
| Categorical variables | | | **N** | **%** | | **N** | | **%** | | | **N** | | **%** | |
| Gender | Male | | 2690 | 26.9 | | 1345 | | 25.79 | | | 1382 | | 34.03 | |
|  | Female | | 7268 | 72.67 | | 3851 | | 73.84 | | | 2666 | | 65.65 | |
|  | Missing | | 43 | 0.43 | | 19 | | 0.36 | | | 13 | | 0.32 | |
| Ethnicity | White | | 6563 | 65.62 | | 2614 | | 50.12 | | | 2268 | | 55.85 | |
|  | Mixed | | 777 | 7.77 | | 446 | | 8.55 | | | 388 | | 9.55 | |
|  | Asian | | 944 | 9.44 | | 946 | | 18.14 | | | 451 | | 11.11 | |
|  | Black | | 1033 | 10.33 | | 628 | | 12.04 | | | 629 | | 15.49 | |
|  | Chinese | | 75 | 0.75 | | 118 | | 2.26 | | | 15 | | 0.37 | |
|  | Other | | 219 | 2.19 | | 218 | | 4.18 | | | 160 | | 3.94 | |
|  | Missing | | 390 | 3.9 | | 245 | | 4.7 | | | 150 | | 3.69 | |
| IMD Decile | 1 | | 849 | 8.49 | | 435 | | 8.34 | | | 549 | | 13.52 | |
|  | 2 | | 2696 | 26.96 | | 1361 | | 26.1 | | | 1267 | | 31.2 | |
|  | 3 | | 2096 | 20.96 | | 1063 | | 20.38 | | | 845 | | 20.81 | |
|  | 4 | | 1258 | 12.58 | | 644 | | 12.35 | | | 433 | | 10.66 | |
|  | 5 | | 886 | 8.86 | | 571 | | 10.95 | | | 316 | | 7.78 | |
|  | 6 | | 790 | 7.9 | | 411 | | 7.88 | | | 242 | | 5.96 | |
|  | 7 | | 493 | 4.93 | | 288 | | 5.52 | | | 141 | | 3.47 | |
|  | 8 | | 484 | 4.84 | | 209 | | 4.01 | | | 132 | | 3.25 | |
|  | 9 | | 203 | 2 | | 106 | | 2.03 | | | 61 | | 1.5 | |
|  | 10 | | 85 | 1 | | 47 | | 0.9 | | | 18 | | 0.44 | |
|  | Missing | | 161 | 2 | | 80 | | 1.53 | | | 57 | | 1.4 | |
| Sexual orientation | Heterosexual | | 6650 | 66.49 | | 3604 | | 69.11 | | | 2851 | | 70.2 | |
|  | Gay/Lesbian | | 308 | 3.08 | | 177 | | 3.39 | | | 103 | | 2.54 | |
|  | Bi-sexual | | 449 | 4.49 | | 302 | | 5.79 | | | 141 | | 3.47 | |
|  | Missing | | 2594 | 25.94 | | 1132 | | 21.71 | | | 966 | | 23.79 | |
| Medication | Prescribed not taking | | 432 | 4.32 | | 241 | | 4.62 | | | 194 | | 4.78 | |
|  | Prescribed and taking | | 2457 | 24.57 | | 1275 | | 24.45 | | | 1284 | | 31.62 | |
|  | Not prescribed | | 6474 | 64.73 | | 3406 | | 65.31 | | | 2290 | | 56.39 | |
|  | Missing | | 638 | 6.38 | | 293 | | 5.62 | | | 293 | | 7.21 | |
| Long term condition | No | | 6708 | 67.07 | | 3523 | | 67.56 | | | 2546 | | 62.69 | |
|  | Yes | | 1386 | 13.86 | | 782 | | 15 | | | 709 | | 17.46 | |
|  | Missing | | 1907 | 19.07 | | 910 | | 17.45 | | | 806 | | 19.85 | |
| Problem descriptor | Depression | | 3882 | 38.82 | | 2072 | | 39.73 | | | 1777 | | 43.76 | |
|  | Mixed A.D | | 564 | 5.64 | | 287 | | 5.5 | | | 288 | | 7.09 | |
|  | GAD | | 1836 | 18.36 | | 843 | | 16.16 | | | 378 | | 9.31 | |
|  | OCD | | 295 | 2.95 | | 199 | | 3.82 | | | 111 | | 2.73 | |
|  | PTSD | | 256 | 2.56 | | 128 | | 2.45 | | | 281 | | 6.92 | |
|  | Other Phobia & Panic | | 696 | 6.96 | | 308 | | 5.91 | | | 306 | | 7.54 | |
|  | Social Phobia | | 565 | 5.65 | | 352 | | 6.75 | | | 270 | | 6.65 | |
|  | Unspecified anxiety | | 424 | 4.24 | | 231 | | 4.43 | | | 146 | | 3.6 | |
|  | Missing | | 1483 | 14.83 | | 795 | | 15.24 | | | 504 | | 12.41 | |
| Clinical outcomes | Reliable recovery | | 5,200 | 52 | | 2396 | | 45.94 | | | 1506 | | 37.08 | |
|  | Reliable improvement | | 7587 | 75.86 | | 3735 | | 71.62 | | | 2614 | | 64.37 | |
|  | Deterioration | | 564 | 5.64 | | 339 | | 6.5 | | | 369 | | 9.09 | |
|  | Attrition | | 2697 | 28.93 | | 1486 | | 30.72 | | | 1508 | | 40.69 | |
| ^a^ baseline variables were only used in sensitivity analyses to compute RI-CLPMs | | | | | | | | | | | | | | |
| NEET: Not in employment, education or training. PHQ9: Patient Health Questionnaire. GAD7: Generalized Anxiety Disorder Questionnaire. A.D: Anxiety and Depression WSAS: Work and Social Adjustment Scale. LI: Low intensity. HI: High intensity. A.D. Anxiety and Depression. GAD: Generalized Anxiety Disorder. OCD: Obsessive Compulsive Disorder. PTSD: Post-Traumatic Stress Disorder | | | | | | | | | | | | | | |
