# Supplementary material for: The bi-directional influence of social functioning and mental health symptoms during psychological treatment: A cross-lagged analysis in young adults
Source: Int J Clin Health Psychol. 2025 Jul 5;25(3):100608. doi: 10.1016/j.ijchp.2025.100608 (PMC12272429; doi:10.1016/j.ijchp.2025.100608)
Supplement: Supplementary file 3 [file mmc3.pdf]

Results: Associations between depressive symptoms and impairment in participation in social leisure activities

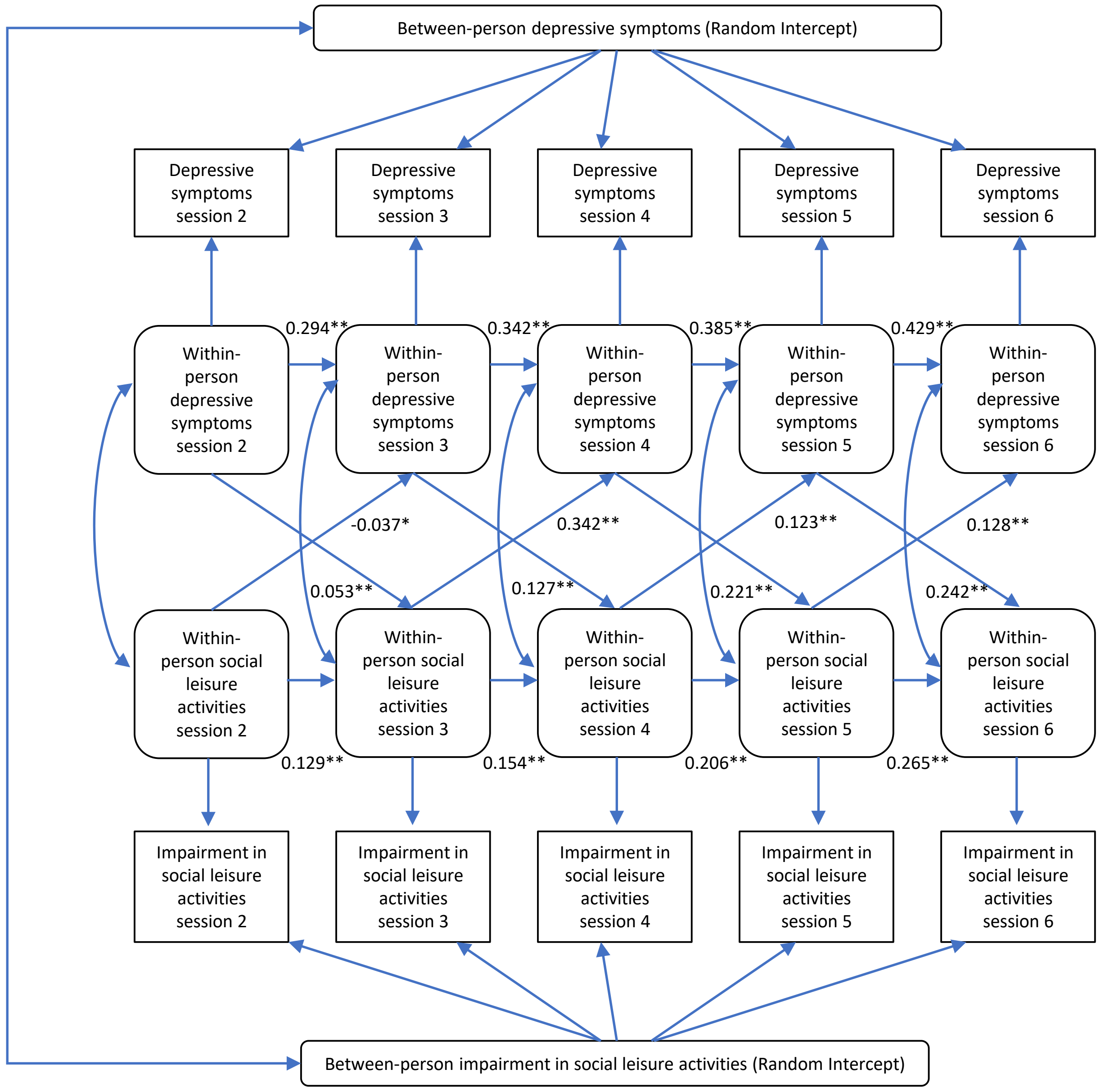

** <.001

* <.01

Results: Associations between anxiety symptoms and impairment in participation in social leisure activities

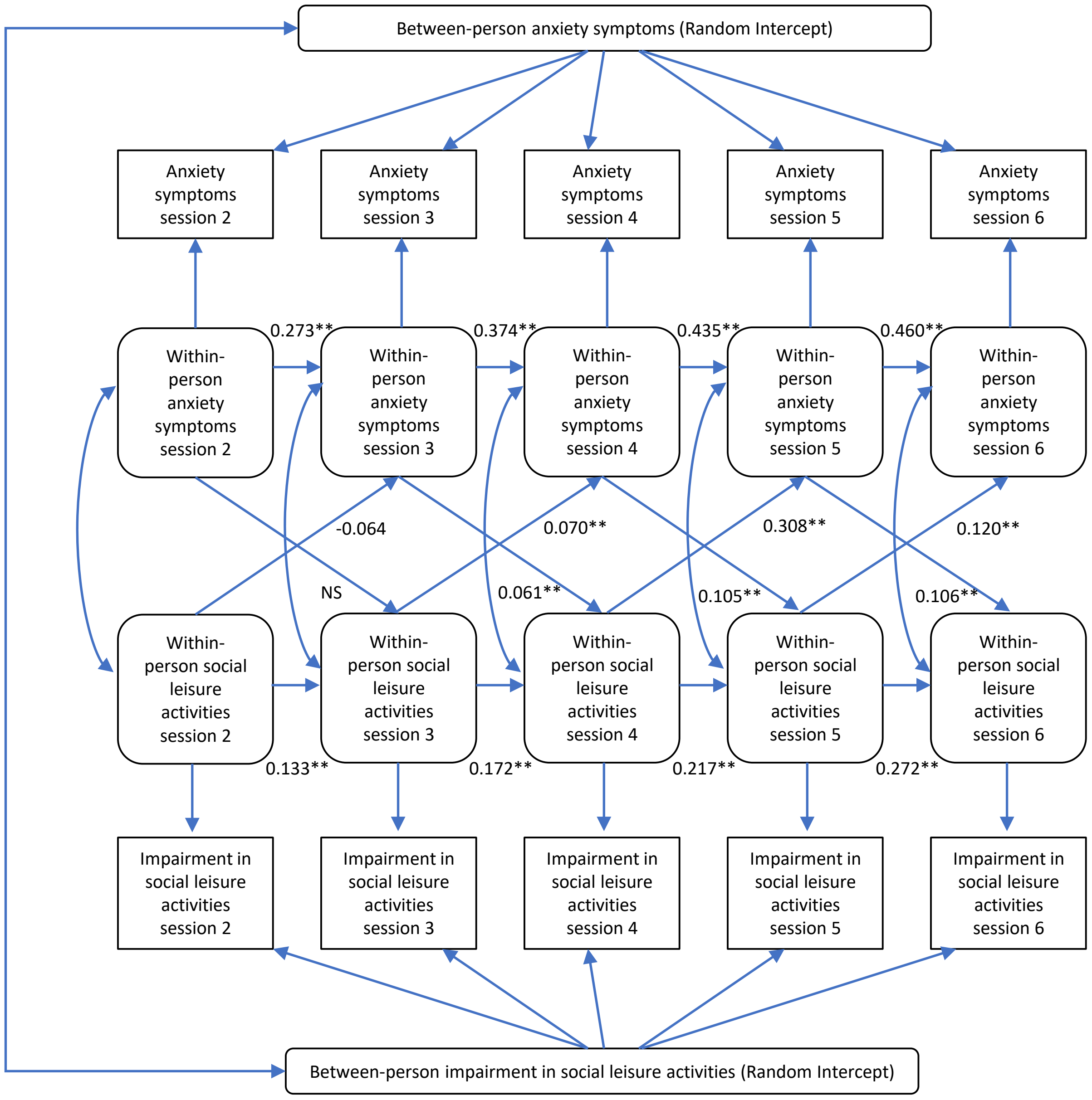

**: <.001

*: <.01

NS: Not significant

Results: Associations between depressive symptoms and impairment in forming close social relationships

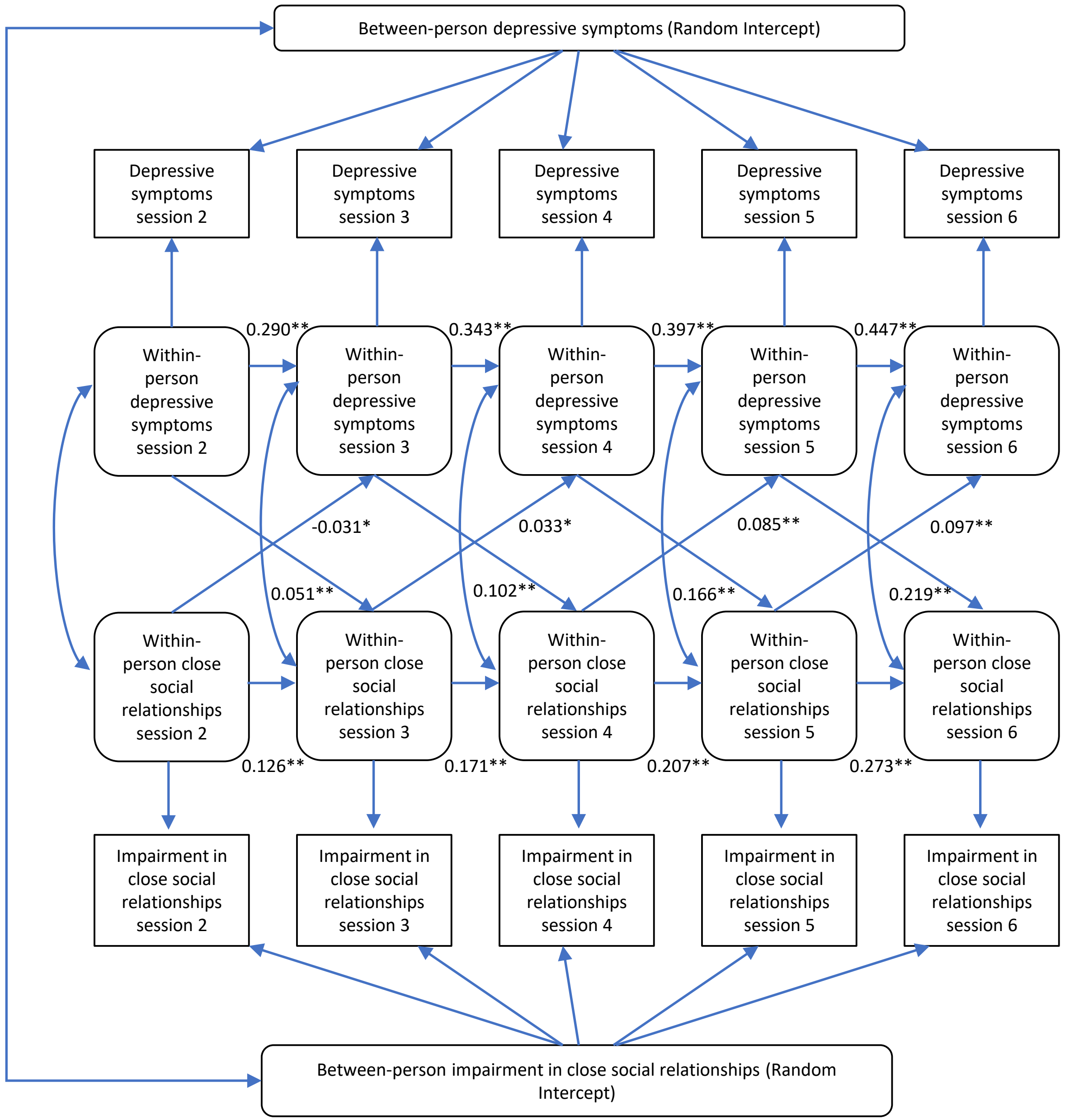

** <.001
* <.01

Results: Associations between anxiety symptoms and impairment in forming close social relationships

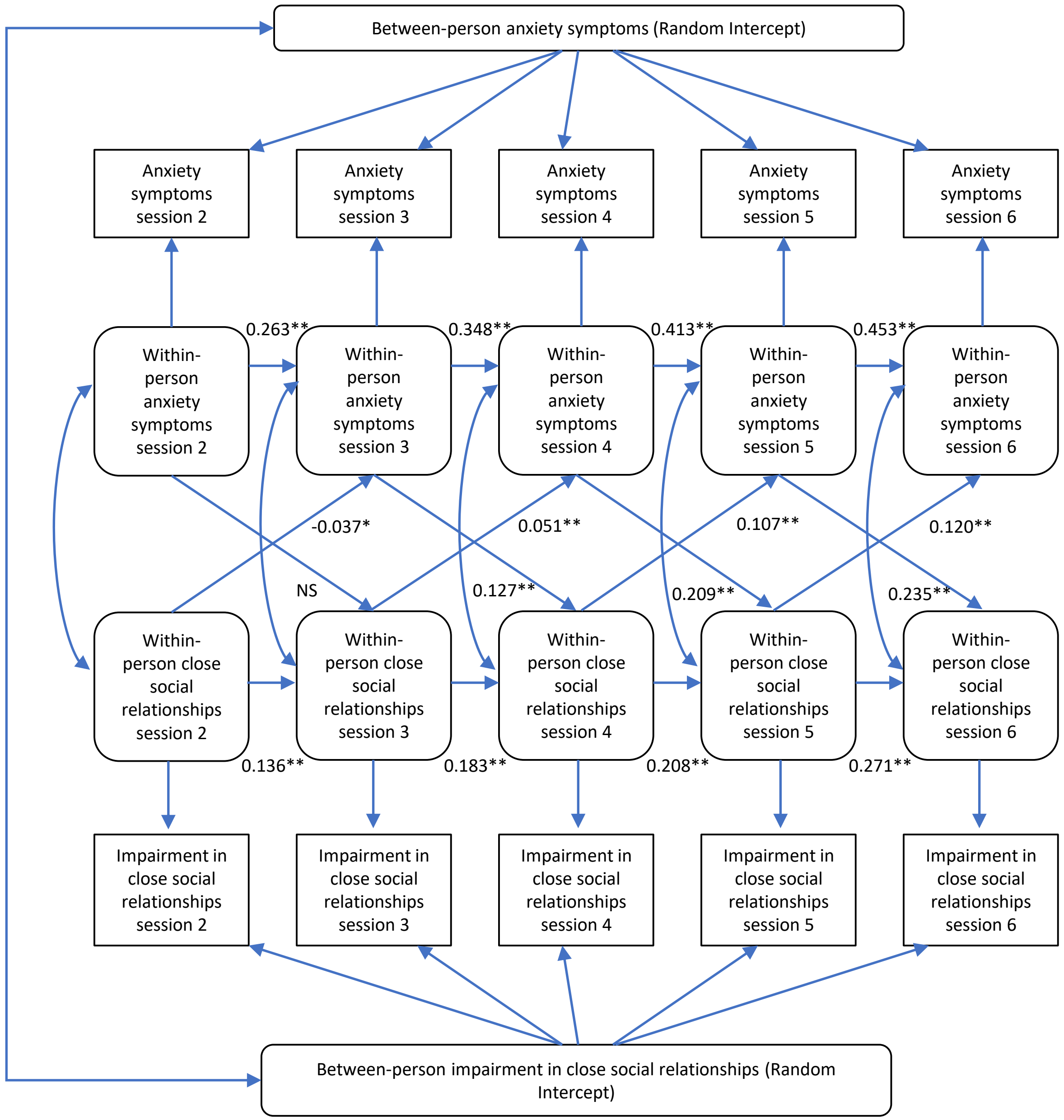

**: <.001
*: <.01
NS: Not significant
