# Supplementary material for: The bi-directional influence of social functioning and mental health symptoms during psychological treatment: A cross-lagged analysis in young adults
Source: Int J Clin Health Psychol. 2025 Jul 5;25(3):100608. doi: 10.1016/j.ijchp.2025.100608 (PMC12272429; doi:10.1016/j.ijchp.2025.100608)
Supplement: Supplementary file 4 [file mmc4.docx]

# Appendix 4: Sensitivity analysis model fit

1. Covariates added

| Model | RMSEA | CFI | TFI | SRMR |
| --- | --- | --- | --- | --- |
| 1 (PHQ, WSAS3) | 0.04 | 0.995 | 0.931 | 0.008 |
| 2 (GAD, WSAS3) | 0.041 | 0.994 | 0.924 | 0.009 |
| 3 (PHQ, WSAS5) | 0.043 | 0.994 | 0.921 | 0.009 |
| 4 (GAD, WSAS5) | 0.043 | 0.993 | 0.913 | 0.01 |
| RMSEA: Root Mean Square Error of Approximation, CFI: Comparative Fit Index, TLI: Tucker-Lewis Index, SRMR: Standardized Root Mean squared Residual, PHQ: Patient Health Questionnaire, GAD: General Anxiety Disorder Questionnaire, WSAS: Work and Social Adjustment Scale | | | | |

1. Including only those with depression or mixed anxiety and depression as their problem descriptor

| Model | RMSEA | CFI | TFI | SRMR |
| --- | --- | --- | --- | --- |
| 1 (PHQ, WSAS3) | 0.037 | 0.993 | 0.984 | 0.022 |
| 3 (PHQ, WSAS5) | 0.038 | 0.992 | 0.983 | 0.024 |
| RMSEA: Root Mean Square Error of Approximation, CFI: Comparative Fit Index, TLI: Tucker-Lewis Index, SRMR: Standardized Root Mean squared Residual, PHQ: Patient Health Questionnaire, WSAS: Work and Social Adjustment Scale | | | | |

1. Including only those with anxiety or mixed anxiety and depression as their problem descriptor

| Model | RMSEA | CFI | TFI | SRMR |
| --- | --- | --- | --- | --- |
| 2 (GAD, WSAS3) | 0.044 | 0.99 | 0.979 | 0.026 |
| 4 (GAD, WSAS5) | 0.043 | 0.991 | 0.98 | 0.026 |
| RMSEA: Root Mean Square Error of Approximation, CFI: Comparative Fit Index, TLI: Tucker-Lewis Index, SRMR: Standardized Root Mean squared Residual, GAD: General Anxiety Disorder Questionnaire, WSAS: Work and Social Adjustment Scale | | | | |

1. Employed

| Model | RMSEA | CFI | TFI | SRMR |
| --- | --- | --- | --- | --- |
| 1 (PHQ, WSAS3) | 0.035 | 0.994 | 0.987 | 0.022 |
| 2 (GAD, WSAS3) | 0.04 | 0.991 | 0.982 | 0.023 |
| 3 (PHQ, WSAS5) | 0.035 | 0.994 | 0.987 | 0.022 |
| 4 (GAD, WSAS5) | 0.04 | 0.991 | 0.982 | 0.025 |
| RMSEA: Root Mean Square Error of Approximation, CFI: Comparative Fit Index, TLI: Tucker-Lewis Index, SRMR: Standardized Root Mean squared Residual, PHQ: Patient Health Questionnaire, GAD: General Anxiety Disorder Questionnaire, WSAS: Work and Social Adjustment Scale | | | | |

1. Students

| Model | RMSEA | CFI | TFI | SRMR |
| --- | --- | --- | --- | --- |
| 1 (PHQ, WSAS3) | 0.038 | 0.993 | 0.985 | 0.023 |
| 2 (GAD, WSAS3) | 0.039 | 0.992 | 0.983 | 0.024 |
| 3 (PHQ, WSAS5) | 0.038 | 0.993 | 0.985 | 0.024 |
| 4 (GAD, WSAS5) | 0.037 | 0.993 | 0.985 | 0.022 |
| RMSEA: Root Mean Square Error of Approximation, CFI: Comparative Fit Index, TLI: Tucker-Lewis Index, SRMR: Standardized Root Mean squared Residual, PHQ: Patient Health Questionnaire, GAD: General Anxiety Disorder Questionnaire, WSAS: Work and Social Adjustment Scale | | | | |

1. NEET (Not in Employment, Education or Training)

| Model | RMSEA | CFI | TFI | SRMR |
| --- | --- | --- | --- | --- |
| 1 (PHQ, WSAS3) | 0.034 | 0.994 | 0.987 | 0.02 |
| 2 (GAD, WSAS3) | 0.04 | 0.991 | 0.981 | 0.024 |
| 3 (PHQ, WSAS5) | 0.037 | 0.992 | 0.984 | 0.024 |
| 4 (GAD, WSAS5) | 0.041 | 0.99 | 0.979 | 0.026 |
| RMSEA: Root Mean Square Error of Approximation, CFI: Comparative Fit Index, TLI: Tucker-Lewis Index, SRMR: Standardized Root Mean squared Residual, PHQ: Patient Health Questionnaire, GAD: General Anxiety Disorder Questionnaire, WSAS: Work and Social Adjustment Scale | | | | |
