# Supplementary material for: The bi-directional influence of social functioning and mental health symptoms during psychological treatment: A cross-lagged analysis in young adults
Source: Int J Clin Health Psychol. 2025 Jul 5;25(3):100608. doi: 10.1016/j.ijchp.2025.100608 (PMC12272429; doi:10.1016/j.ijchp.2025.100608)
Supplement: Supplementary file 5 [file mmc5.docx]

# Appendix 5: Comparison of covariate and unadjusted models

|  |  |  |  | Main analyses (no covariates included) | | Covariates included | |
| --- | --- | --- | --- | --- | --- | --- | --- |
| Model | **Path** | **Predictor** | **Outcome** | **Standardized coefficient** | **p-value** | **Standardized coefficient** | **p-value** |
| 1 (PHQ9, WSAS 3) | Autoregressive | WSAS3 S2 | WSAS3 S3 | 0.129 | <.001 | 0.073 | <.001 |
|  |  | WSAS3 S3 | WSAS3 S4 | 0.154 | <.001 | 0.173 | <.001 |
|  |  | WSAS3 S4 | WSAS3 S5 | 0.206 | <.001 | 0.252 | <.001 |
|  |  | WSAS3 S5 | WSAS3 S6 | 0.265 | <.001 | 0.315 | <.001 |
|  |  | PHQ9 S2 | PHQ9 S3 | 0.294 | <.001 | 0.172 | <.001 |
|  |  | PHQ9 S3 | PHQ9 S4 | 0.342 | <.001 | 0.379 | <.001 |
|  |  | PHQ9 S4 | PHQ9 S5 | 0.385 | <.001 | 0.452 | <.001 |
|  |  | PHQ9 S5 | PHQ9 S6 | 0.429 | <.001 | 0.495 | <.001 |
|  | Cross Lagged | WSAS3 S2 | PHQ9 S3 | -0.037 | 0.003 | -0.025 | 0.041 |
|  |  | WSAS3 S3 | PHQ9 S4 | 0.342 | <.001 | 0.082 | <.001 |
|  |  | WSAS3 S4 | PHQ9 S5 | 0.123 | <.001 | 0.109 | <.001 |
|  |  | WSAS3 S5 | PHQ9 S6 | 0.128 | <.001 | 0.106 | <.001 |
|  |  | PHQ9 S2 | WSAS3 S3 | 0.053 | <.001 | 0.04 | 0.004 |
|  |  | PHQ9 S3 | WSAS3 S4 | 0.127 | <.001 | 0.161 | <.001 |
|  |  | PHQ9 S4 | WSAS3 S5 | 0.221 | <.001 | 0.221 | <.001 |
|  |  | PHQ9 S5 | WSAS3 S6 | 0.242 | <.001 | 0.226 | <.001 |
| 2 (GAD7, WSAS3) | Autoregressive | WSAS3 S2 | WSAS3 S3 | 0.133 | <.001 | 0.075 | <.001 |
|  |  | WSAS3 S3 | WSAS S4 | 0.172 | <.001 | 0.179 | <.001 |
|  |  | WSAS3 S4 | WSAS S5 | 0.217 | <.001 | 0.258 | <.001 |
|  |  | WSAS3 S5 | WSAS S6 | 0.272 | <.001 | 0.328 | <.001 |
|  |  | GAD7 S2 | GAD7 S3 | 0.273 | <.001 | 0.185 | <.001 |
|  |  | GAD7S3 | GAD7 S4 | 0.374 | <.001 | 0.363 | <.001 |
|  |  | GAD7 S4 | GAD7 S5 | 0.435 | <.001 | 0.448 | <.001 |
|  |  | GAD7 S5 | GAD7 S6 | 0.460 | <.001 | 0.481 | <.001 |
|  | Cross Lagged | WSAS3 S2 | GAD7 S3 | -0.064 | 0.015 | -0.019 | 0.096 |
|  |  | WSAS3 S3 | GAD7 S4 | 0.170 | <.001 | 0.078 | <.001 |
|  |  | WSAS3 S4 | GAD7 S5 | 0.308 | <.001 | 0.089 | <.001 |
|  |  | WSAS3 S5 | GAD7 S6 | 0.358 | <.001 | 0.108 | <.001 |
|  |  | GAD7 S2 | WSAS3 S3 | 0.007 | 0.313 | 0.032 | 0.014 |
|  |  | GAD7S3 | WSAS3 S4 | 0.061 | <.001 | 0.152 | <.001 |
|  |  | GAD7 S4 | WSAS3 S5 | 0.105 | <.001 | 0.212 | <.001 |
|  |  | GAD7 S5 | WSAS3 S6 | 0.106 | <.001 | 0.214 | <.001 |
| 3 (PHQ9, WSAS5) | Autoregressive | WSAS5 S2 | WSAS5 S3 | 0.126 | <.001 | 0.066 | <.001 |
|  |  | WSAS5 S3 | WSAS5 S4 | 0.171 | <.001 | 0.201 | <.001 |
|  |  | WSAS5 S4 | WSAS5 S5 | 0.207 | <.001 | 0.263 | <.001 |
|  |  | WSAS5 S5 | WSAS5 S6 | 0.273 | <.001 | 0.326 | <.001 |
|  |  | PHQ9 S2 | PHQ9 S3 | 0.290 | <.001 | 0.167 | <.001 |
|  |  | PHQ9 S3 | PHQ9 S4 | 0.343 | <.001 | 0.386 | <.001 |
|  |  | PHQ9 S4 | PHQ9 S5 | 0.397 | <.001 | 0.462 | <.001 |
|  |  | PHQ9 S5 | PHQ9 S6 | 0.447 | <.001 | 0.504 | <.001 |
|  | Cross Lagged | WSAS5 S2 | PHQ9 S3 | -0.031 | 0.008 | -0.031 | 0.010 |
|  |  | WSAS5 S3 | PHQ9 S4 | 0.033 | 0.009 | 0.060 | <.001 |
|  |  | WSAS5 S4 | PHQ9 S5 | 0.085 | <.001 | 0.088 | <.001 |
|  |  | WSAS5 S5 | PHQ9 S6 | 0.097 | <.001 | 0.094 | <.001 |
|  |  | PHQ9 S2 | WSAS5 S3 | 0.051 | <.001 | 0.021 | 0.136 |
|  |  | PHQ9 S3 | WSAS5 S4 | 0.102 | <.001 | 0.140 | <.001 |
|  |  | PHQ9 S4 | WSAS5 S5 | 0.166 | <.001 | 0.181 | <.001 |
|  |  | PHQ9 S5 | WSAS5 S6 | 0.219 | <.001 | 0.214 | <.001 |
| 4 (GAD7, WSAS5) | Autoregressive | WSAS5 S2 | WSAS5 S3 | 0.136 | <.001 | 0.067 | <.001 |
|  |  | WSAS5 S3 | WSAS S4 | 0.183 | <.001 | 0.198 | <.001 |
|  |  | WSAS5 S4 | WSAS S5 | 0.208 | <.001 | 0.265 | <.001 |
|  |  | WSAS5 S5 | WSAS S6 | 0.271 | <.001 | 0.336 | <.001 |
|  |  | GAD7 S2 | GAD7 S3 | 0.263 | <.001 | 0.183 | <.001 |
|  |  | GAD7S3 | GAD7 S4 | 0.348 | <.001 | 0.367 | <.001 |
|  |  | GAD7 S4 | GAD7 S5 | 0.413 | <.001 | 0.450 | <.001 |
|  |  | GAD7 S5 | GAD7 S6 | 0.453 | <.001 | 0.486 | <.001 |
|  | Cross Lagged | WSAS5 S2 | GAD7 S3 | -0.037 | 0.002 | -0.025 | 0.029 |
|  |  | WSAS5 S3 | GAD7 S4 | 0.051 | <.001 | 0.057 | <.001 |
|  |  | WSAS5 S4 | GAD7 S5 | 0.107 | <.001 | 0.086 | <.001 |
|  |  | WSAS5 S5 | GAD7 S6 | 0.120 | <.001 | 0.098 | <.001 |
|  |  | GAD7 S2 | WSAS5 S3 | 0.013 | 0.357 | 0.018 | 0.166 |
|  |  | GAD7S3 | WSAS5 S4 | 0.127 | <.001 | 0.144 | <.001 |
|  |  | GAD7 S4 | WSAS5 S5 | 0.209 | <.001 | 0.180 | <.001 |
|  |  | GAD7 S5 | WSAS5 S6 | 0.235 | <.001 | 0.189 | <.001 |

PHQ9: Patient Health Questionnaire. WSAS: Work and Social Adjustment Scale. GAD7: General Anxiety Disorder Questionnaire. S: Session
