# Supplementary material for: The bi-directional influence of social functioning and mental health symptoms during psychological treatment: A cross-lagged analysis in young adults
Source: Int J Clin Health Psychol. 2025 Jul 5;25(3):100608. doi: 10.1016/j.ijchp.2025.100608 (PMC12272429; doi:10.1016/j.ijchp.2025.100608)
Supplement: Supplementary file 6 [file mmc6.docx]

# Appendix 6: Sensitivity analysis

## 6.1: Problem descriptor

| Depression or mixed anxiety and depression diagnoses only | | | | | | Anxiety or mixed anxiety and depression diagnoses only | | | | | |
| --- | --- | --- | --- | --- | --- | --- | --- | --- | --- | --- | --- |
| **Model** | **Path** | **Predictor** | **Outcome** | **Standardized coefficient** | **p-value** | **Model** | **Path** | **Predictor** | **Outcome** | **Standardized coefficient** | **p-value** |
| 1 (PHQ9, WSAS 3) | Autoregressive | WSAS3 S2 | WSAS3 S3 | 0.129 | <.001 | 2 (GAD7, WSAS3) | Autoregressive | WSAS3 S2 | WSAS3 S3 | 0.14 | <.001 |
| n=9008 |  | WSAS3 S3 | WSAS3 S4 | 0.179 | <.001 | n=8410 |  | WSAS3 S3 | WSAS S4 | 0.16 | <.001 |
|  |  | WSAS3 S4 | WSAS3 S5 | 0.216 | <.001 |  |  | WSAS3 S4 | WSAS S5 | 0.196 | <.001 |
|  |  | WSAS3 S5 | WSAS3 S6 | 0.272 | <.001 |  |  | WSAS3 S5 | WSAS S6 | 0.273 | <.001 |
|  |  | PHQ9 S2 | PHQ9 S3 | 0.226 | <.001 |  |  | GAD7 S2 | GAD7 S3 | 0.245 | <.001 |
|  |  | PHQ9 S3 | PHQ9 S4 | 0.35 | <.001 |  |  | GAD7S3 | GAD7 S4 | 0.347 | <.001 |
|  |  | PHQ9 S4 | PHQ9 S5 | 0.444 | <.001 |  |  | GAD7 S4 | GAD7 S5 | 0.429 | <.001 |
|  |  | PHQ9 S5 | PHQ9 S6 | 0.498 | <.001 |  |  | GAD7 S5 | GAD7 S6 | 0.453 | <.001 |
|  | Cross Lagged | WSAS3 S2 | PHQ9 S3 | -0.033 | 0.087 |  | Cross Lagged | WSAS3 S2 | GAD7 S3 | -0.009 | 0.62 |
|  |  | WSAS3 S3 | PHQ9 S4 | 0.089 | <.001 |  |  | WSAS3 S3 | GAD7 S4 | 0.062 | 0.001 |
|  |  | WSAS3 S4 | PHQ9 S5 | 0.106 | <.001 |  |  | WSAS3 S4 | GAD7 S5 | 0.117 | <.001 |
|  |  | WSAS3 S5 | PHQ9 S6 | 0.107 | <.001 |  |  | WSAS3 S5 | GAD7 S6 | 0.149 | <.001 |
|  |  | PHQ9 S2 | WSAS3 S3 | 0.037 | 0.086 |  |  | GAD7 S2 | WSAS3 S3 | -0.009 | 0.658 |
|  |  | PHQ9 S3 | WSAS3 S4 | 0.15 | <.001 |  |  | GAD7S3 | WSAS3 S4 | 0.14 | <.001 |
|  |  | PHQ9 S4 | WSAS3 S5 | 0.238 | <.001 |  |  | GAD7 S4 | WSAS3 S5 | 0.256 | <.001 |
|  |  | PHQ9 S5 | WSAS3 S6 | 0.264 | <.001 |  |  | GAD7 S5 | WSAS3 S6 | 0.257 | <.001 |
| 3 (PHQ9, WSAS5) | Autoregressive | WSAS5 S2 | WSAS5 S3 | 0.112 | <.001 | 4 (GAD7, WSAS5) | Autoregressive | WSAS5 S2 | WSAS5 S3 | 0.156 | <.001 |
| n=9008 |  | WSAS5 S3 | WSAS5 S4 | 0.177 | <.001 | n=8410 |  | WSAS5 S3 | WSAS S4 | 0.18 | <.001 |
|  |  | WSAS5 S4 | WSAS5 S5 | 0.217 | <.001 |  |  | WSAS5 S4 | WSAS S5 | 0.178 | <.001 |
|  |  | WSAS5 S5 | WSAS5 S6 | 0.282 | <.001 |  |  | WSAS5 S5 | WSAS S6 | 0.244 | <.001 |
|  |  | PHQ9 S2 | PHQ9 S3 | 0.226 | <.001 |  |  | GAD7 S2 | GAD7 S3 | 0.247 | <.001 |
|  |  | PHQ9 S3 | PHQ9 S4 | 0.363 | <.001 |  |  | GAD7S3 | GAD7 S4 | 0.341 | <.001 |
|  |  | PHQ9 S4 | PHQ9 S5 | 0.455 | <.001 |  |  | GAD7 S4 | GAD7 S5 | 0.441 | <.001 |
|  |  | PHQ9 S5 | PHQ9 S6 | 0.502 | <.001 |  |  | GAD7 S5 | GAD7 S6 | 0.479 | <.001 |
|  | Cross Lagged | WSAS5 S2 | PHQ9 S3 | -0.04 | 0.027 |  | Cross Lagged | WSAS5 S2 | GAD7 S3 | -0.015 | 0.412 |
|  |  | WSAS5 S3 | PHQ9 S4 | 0.056 | <.001 |  |  | WSAS5 S3 | GAD7 S4 | 0.054 | 0.003 |
|  |  | WSAS5 S4 | PHQ9 S5 | 0.086 | <.001 |  |  | WSAS5 S4 | GAD7 S5 | 0.09 | <.001 |
|  |  | WSAS5 S5 | PHQ9 S6 | 0.106 | <.001 |  |  | WSAS5 S5 | GAD7 S6 | 0.098 | <.001 |
|  |  | PHQ9 S2 | WSAS5 S3 | 0.015 | 0.493 |  |  | GAD7 S2 | WSAS5 S3 | 0.035 | 0.082 |
|  |  | PHQ9 S3 | WSAS5 S4 | 0.138 | <.001 |  |  | GAD7S3 | WSAS5 S4 | 0.121 | <.001 |
|  |  | PHQ9 S4 | WSAS5 S5 | 0.212 | <.001 |  |  | GAD7 S4 | WSAS5 S5 | 0.203 | <.001 |
|  |  | PHQ9 S5 | WSAS5 S6 | 0.259 | <.001 |  |  | GAD7 S5 | WSAS5 S6 | 0.25 | <.001 |

PHQ9: Patient Health Questionnaire. GAD7: Generalized Anxiety Disorder Questionnaire. WSAS: Work and Social Adjustment Scale. S: Session

## 6.2: Employment status

|  | |  | |  | |  | | Main analyses | | Employed | | Students | | NEET | |
| --- | --- | --- | --- | --- | --- | --- | --- | --- | --- | --- | --- | --- | --- | --- | --- |
| **Model** | **Path** | | **Predictor** | **Outcome** | **Standardized coefficient** | | **p-value** | **Standardized coefficient** | **p-value** | | **Standardized coefficient** | **p-value** | **Standardized coefficient** | | **p-value** |
| 1 (PHQ9, WSAS 3) | Autoregressive | | WSAS3 S2 | WSAS3 S3 | 0.129 | | <.001 | 0.177 | <.001 | | 0.119 | <.001 | 0.043 | | 0.218 |
| n=19600 |  | | WSAS3 S3 | WSAS3 S4 | 0.154 | | <.001 | 0.154 | <.001 | | 0.18 | <.001 | 0.132 | | <.001 |
|  |  | | WSAS3 S4 | WSAS3 S5 | 0.206 | | <.001 | 0.216 | <.001 | | 0.182 | <.001 | 0.205 | | <.001 |
|  |  | | WSAS3 S5 | WSAS3 S6 | 0.265 | | <.001 | 0.245 | <.001 | | 0.272 | <.001 | 0.306 | | <.001 |
|  |  | | PHQ9 S2 | PHQ9 S3 | 0.294 | | <.001 | 0.334 | <.001 | | 0.307 | <.001 | 0.182 | | <.001 |
|  |  | | PHQ9 S3 | PHQ9 S4 | 0.342 | | <.001 | 0.341 | <.001 | | 0.35 | <.001 | 0.358 | | <.001 |
|  |  | | PHQ9 S4 | PHQ9 S5 | 0.385 | | <.001 | 0.371 | <.001 | | 0.376 | <.001 | 0.437 | | <.001 |
|  |  | | PHQ9 S5 | PHQ9 S6 | 0.429 | | <.001 | 0.392 | <.001 | | 0.411 | <.001 | 0.538 | | <.001 |
|  | Cross Lagged | | WSAS3 S2 | PHQ9 S3 | -0.037 | | 0.003 | -0.032 | 0.058 | | -0.021 | 0.365 | -0.073 | | 0.023 |
|  |  | | WSAS3 S3 | PHQ9 S4 | 0.342 | | <.001 | 0.053 | 0.002 | | 0.082 | 0.001 | 0.072 | | 0.004 |
|  |  | | WSAS3 S4 | PHQ9 S5 | 0.123 | | <.001 | 0.121 | <.001 | | 0.112 | <.001 | 0.135 | | <.001 |
|  |  | | WSAS3 S5 | PHQ9 S6 | 0.128 | | <.001 | 0.114 | <.001 | | 0.164 | <.001 | 0.103 | | <.001 |
|  |  | | PHQ9 S2 | WSAS3 S3 | 0.053 | | <.001 | 0.057 | 0.002 | | 0.096 | <.001 | -0.021 | | 0.55 |
|  |  | | PHQ9 S3 | WSAS3 S4 | 0.127 | | <.001 | 0.124 | <.001 | | 0.134 | <.001 | 0.146 | | <.001 |
|  |  | | PHQ9 S4 | WSAS3 S5 | 0.221 | | <.001 | 0.208 | <.001 | | 0.265 | <.001 | 0.21 | | <.001 |
|  |  | | PHQ9 S5 | WSAS3 S6 | 0.242 | | <.001 | 0.24 | <.001 | | 0.231 | <.001 | 0.254 | | <.001 |
| 2 (GAD7, WSAS3) | Autoregressive | | WSAS3 S2 | WSAS3 S3 | 0.133 | | <.001 | 0.185 | <.001 | | 0.14 | <.001 | 0.039 | | 0.27 |
| n=19600 |  | | WSAS3 S3 | WSAS S4 | 0.172 | | <.001 | 0.165 | <.001 | | 0.205 | <.001 | 0.158 | | <.001 |
|  |  | | WSAS3 S4 | WSAS S5 | 0.217 | | <.001 | 0.213 | <.001 | | 0.199 | <.001 | 0.208 | | <.001 |
|  |  | | WSAS3 S5 | WSAS S6 | 0.272 | | <.001 | 0.262 | <.001 | | 0.267 | <.001 | 0.291 | | <.001 |
|  |  | | GAD7 S2 | GAD7 S3 | 0.273 | | <.001 | 0.315 | <.001 | | 0.251 | <.001 | 0.139 | | 0.001 |
|  |  | | GAD7S3 | GAD7 S4 | 0.374 | | <.001 | 0.361 | <.001 | | 0.345 | <.001 | 0.322 | | <.001 |
|  |  | | GAD7 S4 | GAD7 S5 | 0.435 | | <.001 | 0.413 | <.001 | | 0.407 | <.001 | 0.394 | | <.001 |
|  |  | | GAD7 S5 | GAD7 S6 | 0.460 | | <.001 | 0.42 | <.001 | | 0.433 | <.001 | 0.513 | | <.001 |
|  | Cross Lagged | | WSAS3 S2 | GAD7 S3 | -0.064 | | 0.015 | -0.022 | 0.182 | | -0.016 | 0.502 | -0.085 | | 0.009 |
|  |  | | WSAS3 S3 | GAD7 S4 | 0.170 | | <.001 | 0.058 | 0.001 | | 0.094 | <.001 | 0.069 | | 0.007 |
|  |  | | WSAS3 S4 | GAD7 S5 | 0.308 | | <.001 | 0.107 | <.001 | | 0.106 | <.001 | 0.152 | | <.001 |
|  |  | | WSAS3 S5 | GAD7 S6 | 0.358 | | <.001 | 0.124 | <.001 | | 0.164 | <.001 | 0.119 | | <.001 |
|  |  | | GAD7 S2 | WSAS3 S3 | 0.007 | | 0.313 | 0.036 | 0.048 | | 0.039 | 0.147 | -0.056 | | 0.108 |
|  |  | | GAD7S3 | WSAS3 S4 | 0.061 | | <.001 | 0.151 | <.001 | | 0.124 | <.001 | 0.127 | | <.001 |
|  |  | | GAD7 S4 | WSAS3 S5 | 0.105 | | <.001 | 0.241 | <.001 | | 0.263 | <.001 | 0.228 | | <.001 |
|  |  | | GAD7 S5 | WSAS3 S6 | 0.106 | | <.001 | 0.245 | <.001 | | 0.253 | <.001 | 0.287 | | <.001 |
| 3 (PHQ9, WSAS5) | Autoregressive | | WSAS5 S2 | WSAS5 S3 | 0.126 | | <.001 | 0.149 | <.001 | | 0.137 | <.001 | 0.061 | | 0.05 |
| n=19600 |  | | WSAS5 S3 | WSAS5 S4 | 0.171 | | <.001 | 0.162 | <.001 | | 0.185 | <.001 | 0.179 | | <.001 |
|  |  | | WSAS5 S4 | WSAS5 S5 | 0.207 | | <.001 | 0.192 | <.001 | | 0.225 | <.001 | 0.209 | | <.001 |
|  |  | | WSAS5 S5 | WSAS5 S6 | 0.273 | | <.001 | 0.257 | <.001 | | 0.308 | <.001 | 0.263 | | <.001 |
|  |  | | PHQ9 S2 | PHQ9 S3 | 0.290 | | <.001 | 0.323 | <.001 | | 0.301 | <.001 | 0.189 | | <.001 |
|  |  | | PHQ9 S3 | PHQ9 S4 | 0.343 | | <.001 | 0.338 | <.001 | | 0.35 | <.001 | 0.372 | | <.001 |
|  |  | | PHQ9 S4 | PHQ9 S5 | 0.397 | | <.001 | 0.372 | <.001 | | 0.39 | <.001 | 0.467 | | <.001 |
|  |  | | PHQ9 S5 | PHQ9 S6 | 0.447 | | <.001 | 0.402 | <.001 | | 0.441 | <.001 | 0.559 | | <.001 |
|  | Cross Lagged | | WSAS5 S2 | PHQ9 S3 | -0.031 | | 0.008 | 0.004 | 0.789 | | -0.032 | 0.149 | -0.106 | | <.001 |
|  |  | | WSAS5 S3 | PHQ9 S4 | 0.033 | | 0.009 | 0.014 | 0.421 | | 0.066 | 0.007 | 0.033 | | 0.197 |
|  |  | | WSAS5 S4 | PHQ9 S5 | 0.085 | | <.001 | 0.099 | <.001 | | 0.073 | 0.003 | 0.077 | | 0.002 |
|  |  | | WSAS5 S5 | PHQ9 S6 | 0.097 | | <.001 | 0.088 | <.001 | | 0.114 | <.001 | 0.069 | | 0.008 |
|  |  | | PHQ9 S2 | WSAS5 S3 | 0.051 | | <.001 | 0.066 | <.001 | | 0.087 | 0.001 | -0.007 | | 0.835 |
|  |  | | PHQ9 S3 | WSAS5 S4 | 0.102 | | <.001 | 0.096 | <.001 | | 0.113 | <.001 | 0.123 | | <.001 |
|  |  | | PHQ9 S4 | WSAS5 S5 | 0.166 | | <.001 | 0.135 | <.001 | | 0.193 | <.001 | 0.194 | | <.001 |
|  |  | | PHQ9 S5 | WSAS5 S6 | 0.219 | | <.001 | 0.212 | <.001 | | 0.212 | <.001 | 0.234 | | <.001 |
| 4 (GAD7, WSAS5) | Autoregressive | | WSAS5 S2 | WSAS5 S3 | 0.136 | | <.001 | 0.161 | <.001 | | 0.155 | <.001 | 0.058 | | 0.068 |
| n=19600 |  | | WSAS5 S3 | WSAS S4 | 0.183 | | <.001 | 0.171 | <.001 | | 0.203 | <.001 | 0.181 | | <.001 |
|  |  | | WSAS5 S4 | WSAS S5 | 0.208 | | <.001 | 0.193 | <.001 | | 0.22 | <.001 | 0.206 | | <.001 |
|  |  | | WSAS5 S5 | WSAS S6 | 0.271 | | <.001 | 0.265 | <.001 | | 0.302 | <.001 | 0.245 | | <.001 |
|  |  | | GAD7 S2 | GAD7 S3 | 0.263 | | <.001 | 0.311 | <.001 | | 0.252 | <.001 | 0.143 | | <.001 |
|  |  | | GAD7S3 | GAD7 S4 | 0.348 | | <.001 | 0.355 | <.001 | | 0.349 | <.001 | 0.328 | | <.001 |
|  |  | | GAD7 S4 | GAD7 S5 | 0.413 | | <.001 | 0.409 | <.001 | | 0.409 | <.001 | 0.413 | | <.001 |
|  |  | | GAD7 S5 | GAD7 S6 | 0.453 | | <.001 | 0.421 | <.001 | | 0.445 | <.001 | 0.537 | | <.001 |
|  | Cross Lagged | | WSAS5 S2 | GAD7 S3 | -0.037 | | 0.002 | -0.018 | 0.248 | | -0.029 | 0.217 | -0.106 | | <.001 |
|  |  | | WSAS5 S3 | GAD7 S4 | 0.051 | | <.001 | 0.033 | 0.047 | | 0.083 | <.001 | 0.042 | | 0.104 |
|  |  | | WSAS5 S4 | GAD7 S5 | 0.107 | | <.001 | 0.109 | <.001 | | 0.097 | <.001 | 0.12 | | <.001 |
|  |  | | WSAS5 S5 | GAD7 S6 | 0.120 | | <.001 | 0.113 | <.001 | | 0.14 | <.001 | 0.077 | | 0.003 |
|  |  | | GAD7 S2 | WSAS5 S3 | 0.013 | | 0.357 | 0.026 | 0.153 | | 0.032 | 0.229 | -0.041 | | 0.216 |
|  |  | | GAD7S3 | WSAS5 S4 | 0.127 | | <.001 | 0.123 | <.001 | | 0.123 | <.001 | 0.141 | | <.001 |
|  |  | | GAD7 S4 | WSAS5 S5 | 0.209 | | <.001 | 0.181 | <.001 | | 0.248 | <.001 | 0.209 | | <.001 |
|  |  | | GAD7 S5 | WSAS5 S6 | 0.235 | | <.001 | 0.216 | <.001 | | 0.225 | <.001 | 0.28 | | <.001 |

PHQ9: Patient Health Questionnaire. GAD7: Generalized Anxiety Disorder Questionnaire. WSAS: Work and Social Adjustment Scale. NEET: Not in Employment, Education or Training. S: Session
