# Supplementary material for: The bi-directional influence of social functioning and mental health symptoms during psychological treatment: A cross-lagged analysis in young adults
Source: Int J Clin Health Psychol. 2025 Jul 5;25(3):100608. doi: 10.1016/j.ijchp.2025.100608 (PMC12272429; doi:10.1016/j.ijchp.2025.100608)
Supplement: Supplementary file 7 [file mmc7.pptx]

## Slide 1
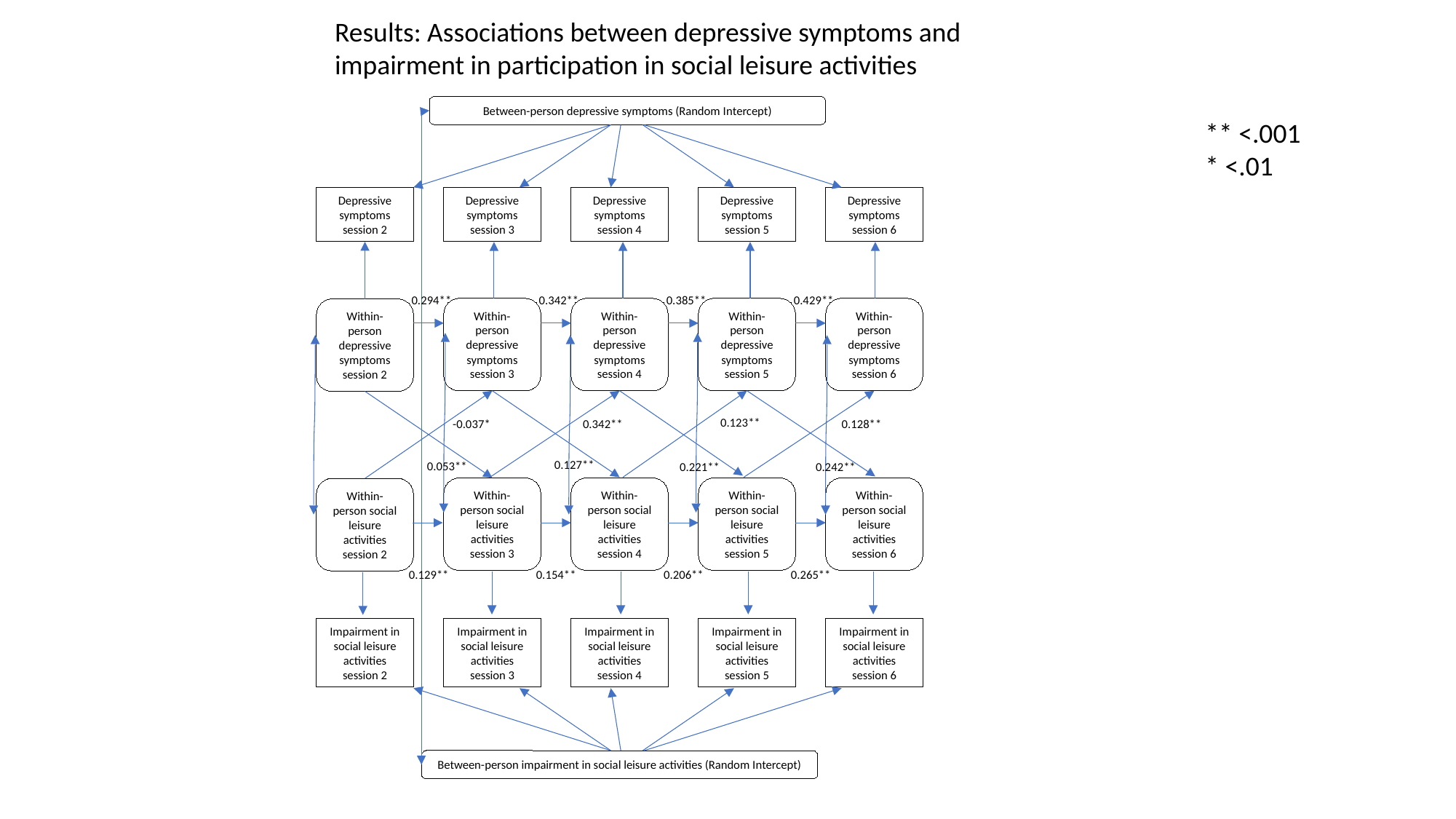

Results: Associations between depressive symptoms and impairment in participation in social leisure activities
Between-person depressive symptoms (Random Intercept)
** <.001
* <.01
Depressive symptoms session 3
Depressive symptoms session 4
Depressive symptoms session 5
Depressive symptoms session 6
Depressive symptoms session 2
0.342**
0.429**
0.294**
0.385**
Within-person depressive symptoms session 6
Within-person depressive symptoms session 4
Within-person depressive symptoms session 5
Within-person depressive symptoms session 3
Within-person depressive symptoms session 2
0.123**
0.342**
-0.037*
0.128**
0.127**
0.053**
0.221**
0.242**
Within-person social leisure activities session 6
Within-person social leisure activities session 4
Within-person social leisure activities session 5
Within-person social leisure activities session 3
Within-person social leisure activities session 2
0.154**
0.265**
0.129**
0.206**
Impairment in social leisure activities session 3
Impairment in social leisure activities session 4
Impairment in social leisure activities session 5
Impairment in social leisure activities session 6
Impairment in social leisure activities session 2
Between-person impairment in social leisure activities (Random Intercept)

## Slide 2
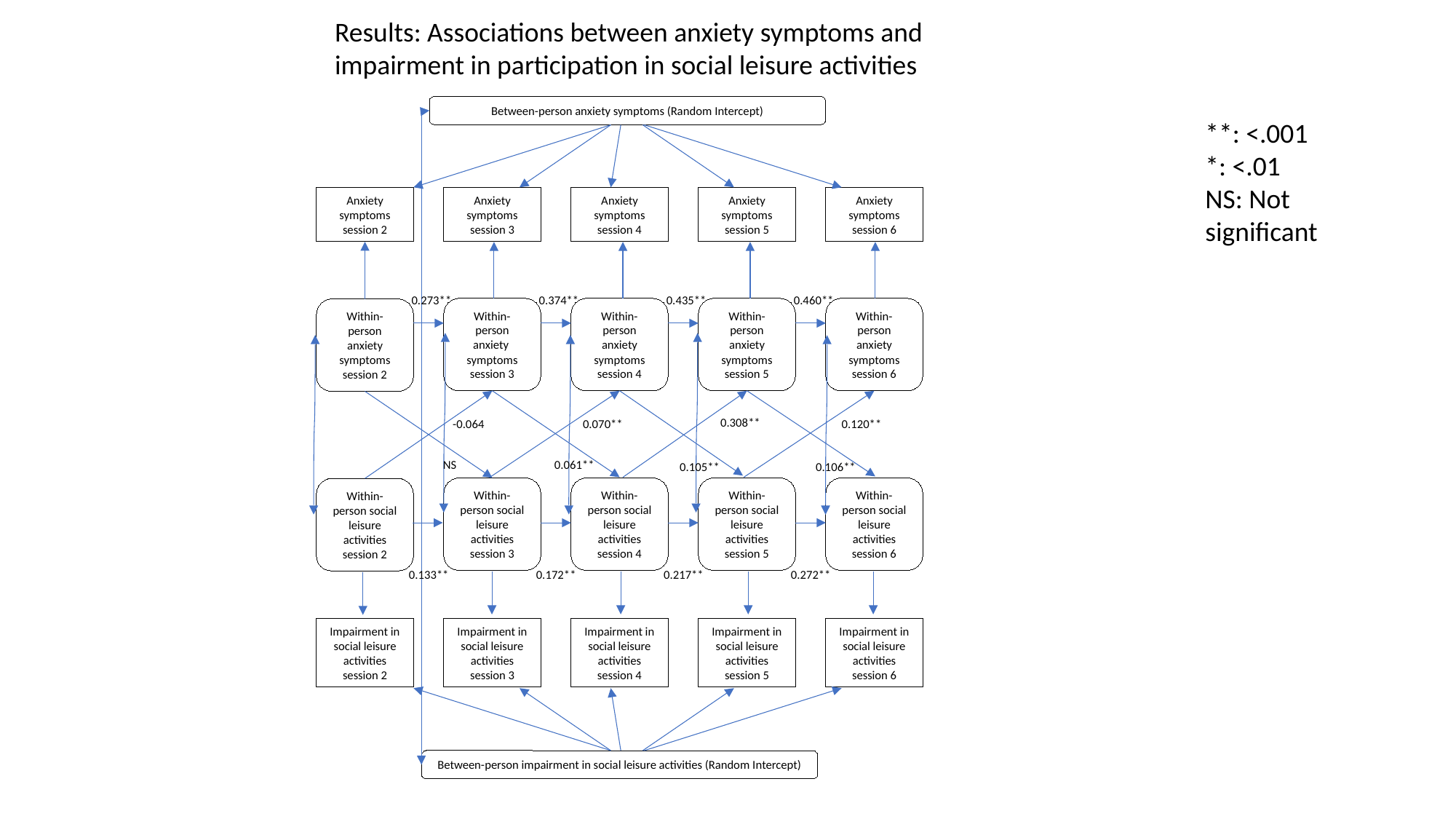

Results: Associations between anxiety symptoms and impairment in participation in social leisure activities
Between-person anxiety symptoms (Random Intercept)
**: <.001
*: <.01
NS: Not significant
Anxiety symptoms session 3
Anxiety symptoms session 4
Anxiety symptoms session 5
Anxiety symptoms session 6
Anxiety symptoms session 2
0.374**
0.460**
0.273**
0.435**
Within-person anxiety symptoms session 6
Within-person anxiety symptoms session 4
Within-person anxiety symptoms session 5
Within-person anxiety symptoms session 3
Within-person anxiety symptoms session 2
0.308**
0.070**
-0.064
0.120**
NS
0.061**
0.105**
0.106**
Within-person social leisure activities session 6
Within-person social leisure activities session 4
Within-person social leisure activities session 5
Within-person social leisure activities session 3
Within-person social leisure activities session 2
0.172**
0.272**
0.133**
0.217**
Impairment in social leisure activities session 3
Impairment in social leisure activities session 4
Impairment in social leisure activities session 5
Impairment in social leisure activities session 6
Impairment in social leisure activities session 2
Between-person impairment in social leisure activities (Random Intercept)

## Slide 3
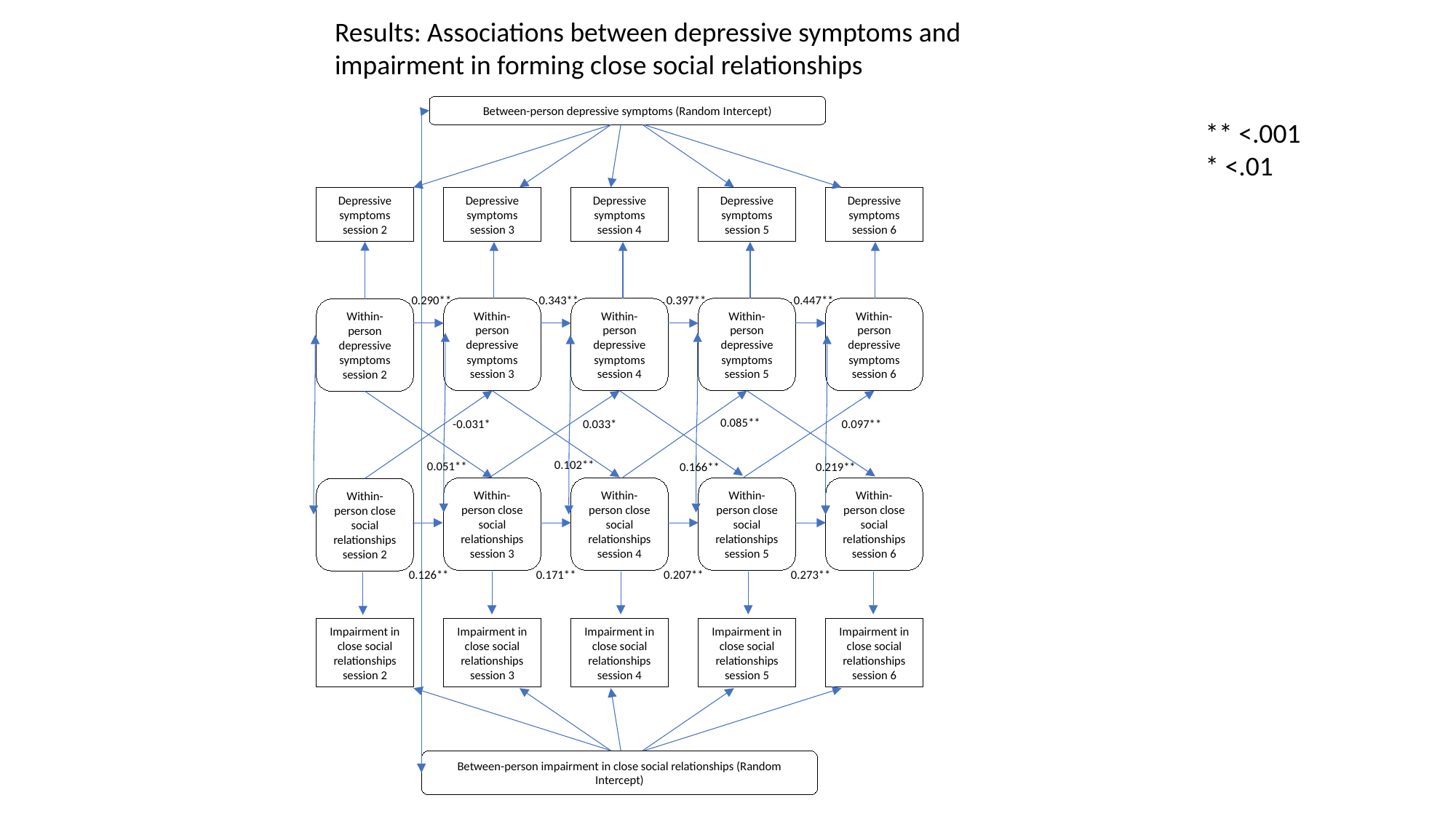

Results: Associations between depressive symptoms and impairment in forming close social relationships
Between-person depressive symptoms (Random Intercept)
** <.001
* <.01
Depressive symptoms session 3
Depressive symptoms session 4
Depressive symptoms session 5
Depressive symptoms session 6
Depressive symptoms session 2
0.343**
0.447**
0.290**
0.397**
Within-person depressive symptoms session 6
Within-person depressive symptoms session 4
Within-person depressive symptoms session 5
Within-person depressive symptoms session 3
Within-person depressive symptoms session 2
0.085**
0.033*
-0.031*
0.097**
0.102**
0.051**
0.166**
0.219**
Within-person close social relationships session 6
Within-person close social relationships session 4
Within-person close social relationships session 5
Within-person close social relationships session 3
Within-person close social relationships session 2
0.171**
0.273**
0.126**
0.207**
Impairment in close social relationships session 3
Impairment in close social relationships session 4
Impairment in close social relationships session 5
Impairment in close social relationships session 6
Impairment in close social relationships session 2
Between-person impairment in close social relationships (Random Intercept)

## Slide 4
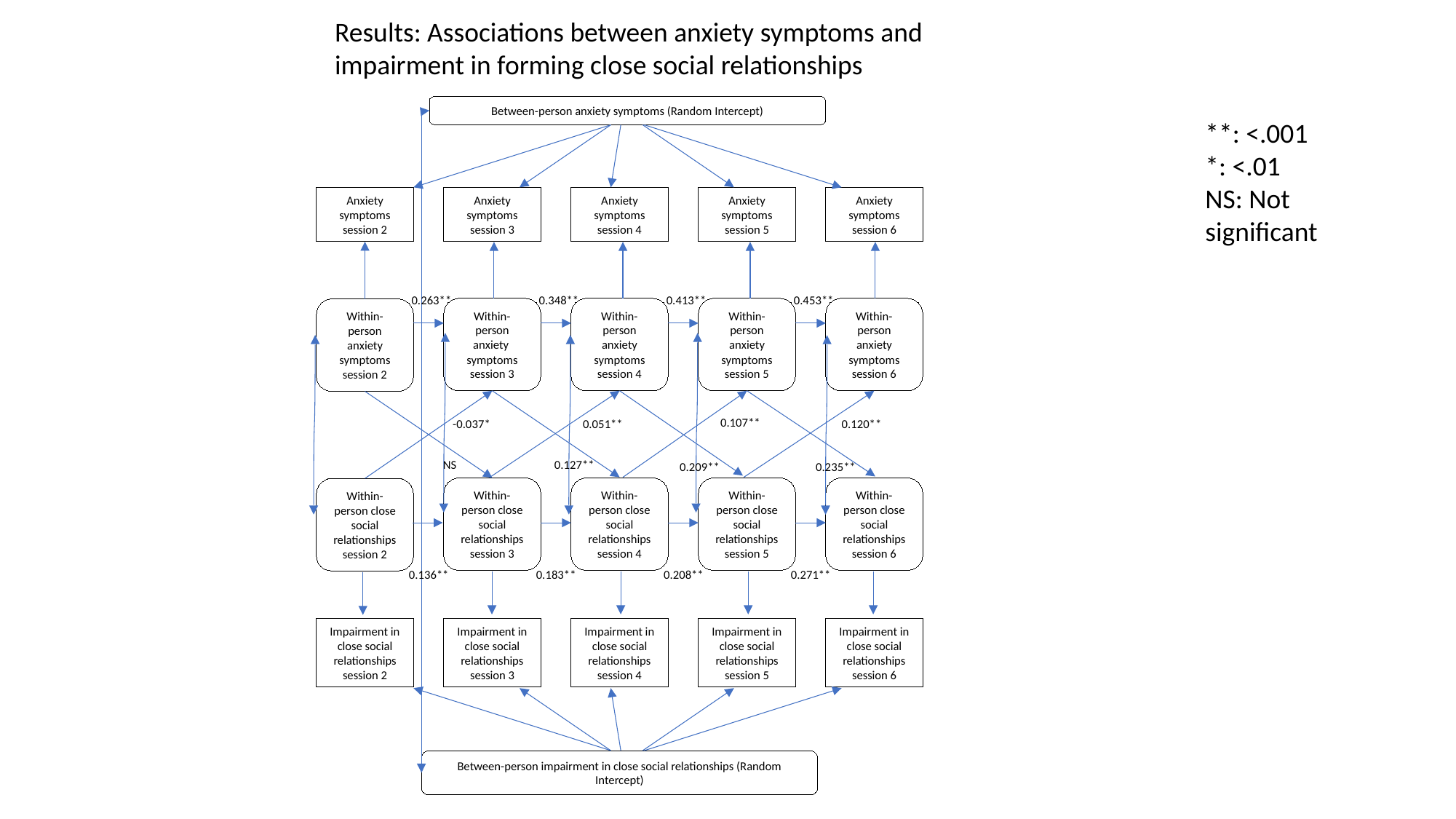

Results: Associations between anxiety symptoms and impairment in forming close social relationships
Between-person anxiety symptoms (Random Intercept)
**: <.001
*: <.01
NS: Not significant
Anxiety symptoms session 3
Anxiety symptoms session 4
Anxiety symptoms session 5
Anxiety symptoms session 6
Anxiety symptoms session 2
0.348**
0.453**
0.263**
0.413**
Within-person anxiety symptoms session 6
Within-person anxiety symptoms session 4
Within-person anxiety symptoms session 5
Within-person anxiety symptoms session 3
Within-person anxiety symptoms session 2
0.107**
0.051**
-0.037*
0.120**
NS
0.127**
0.209**
0.235**
Within-person close social relationships session 6
Within-person close social relationships session 4
Within-person close social relationships session 5
Within-person close social relationships session 3
Within-person close social relationships session 2
0.183**
0.271**
0.136**
0.208**
Impairment in close social relationships session 3
Impairment in close social relationships session 4
Impairment in close social relationships session 5
Impairment in close social relationships session 6
Impairment in close social relationships session 2
Between-person impairment in close social relationships (Random Intercept)
